# Synthesis of 2-Alkenyl-2*H*-indazoles from 2-(2-Carbonylmethyl)-2*H*-indazoles

**DOI:** 10.3390/molecules21020238

**Published:** 2016-02-19

**Authors:** Mei-Huey Lin, Kung-Yu Liang, Chang-Hsien Tsai, Yu-Chun Chen, Hung-Chang Hsiao, Yi-Syuan Li, Chung-Hao Chen, Hau-Chun Wu

**Affiliations:** Department of Chemistry, National Changhua University of Education, Changhua 50007, Taiwan; k7731379@livemail.tw (K.-Y.L.); blueangela39@gmail.com (C.-H.T.); stu01250008@gmail.com (Y.-C.C.); j2261102002@hotmail.com (H.-C.H.); jere035330@hotmail.com (Y.-S.L.); zx25610548zx25610548@yahoo.com.tw (C.-H.C.); smile3156@gmail.com (H.-C.W.)

**Keywords:** indazoles, coumarin, regioselective alkylation, styrene, fluorescence

## Abstract

A procedure has been developed for synthesis of 2-alkenyl-2*H*-indazoles starting from 2-(2-carbonylmethyl)-2*H*-indazoles, which are prepared by gallium/aluminium- and aluminium-mediated, direct, regioselective alkylation of indazoles with α-bromocarbonyl compounds. The structure of 3-(2*H*-indazol-2-yl)-2*H*-chromen-2-one was proven by X-ray crystallography. The styrene- and coumarin-2*H*-indazoles produced by using the new method were found to have interesting fluorescence properties.

## 1. Introduction

Owing to their unique binding affinities to key enzymes and proteins involved metabolic processes, nitrogen-containing heterocyclic compounds have drawn intensive attention in the area of drug discovery [1,2,3,4]. Among these substances, indazoles display a broad range of interesting biological properties that have been used advantageously in the design of new drugs. Consequently, methods for the synthesis of indazole derivatives are in high demand. Special interest has been given to the development of procedures for regioselective introduction of functionality on the two nitrogen atoms of indazoles [5,6,7,8]. This task is simplified somewhat by the fact that the reactivities of the two nitrogen atoms in indazoles are dramatically different. In general, *N*-1 substituted indazoles are thermodynamically favored products of these processes. However, finding new methods for regioselective synthesis of 2*H*-indazoles remains a significant challenge in the field of nitrogen heterocycles. While reasonable progress has been made in devising techniques to prepare 2-alkyl- and 2-aryl-2*H*-indazoles [9,10,11,12,13,14,15,16], fewer advances have been made in developing methods for the preparation of 2-alkenyl-2*H*-indazoles. Thus, a need exists to design operationally simple methods to access the privileged 2-alkenyl-2*H*-indazole pharmacophore.

**Scheme 1:**
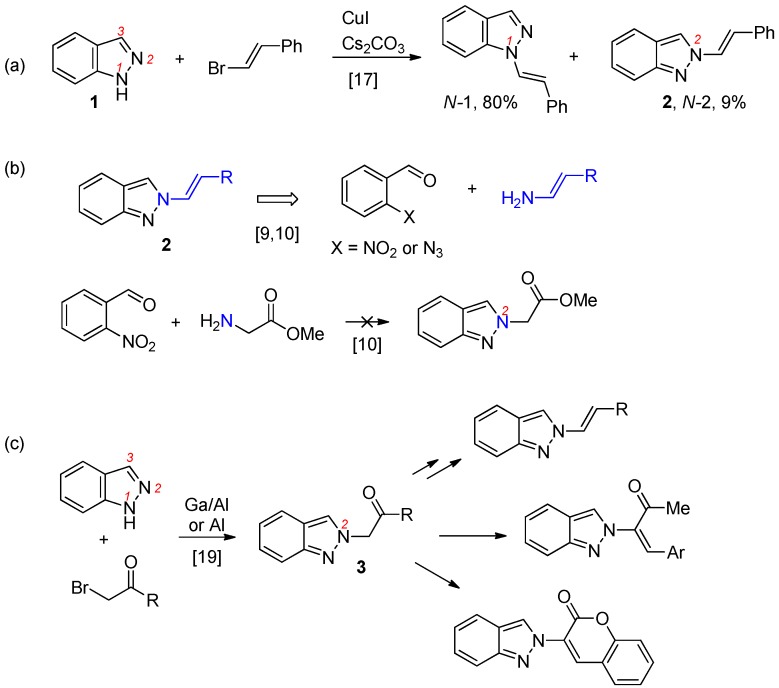
Strategies for the synthesis of 2-alkenyl-2*H*-indazoles.

Of the methods described thus far, copper-catalyzed vinylation reactions appear to hold the most promise as a new 2-alkenyl-2*H*-indazole synthesis approach. Unfortunately, our initial studies of this process demonstrated that reaction of indazole (**1**) with β-bromostyrene is very inefficient, producing **2** in only 9% yield (path (a) in Scheme 1) [17]. An alternative strategy that begins with conventional cyclization reactions of 2-nitrobenzaldehyde and 2-azidobenzaldehyde are not applicable to construction of 2-alkenyl-2*H*-indazoles owing to the substrate limitations associated with *N*-alkenyl primary amine, (path (b), Scheme 1) [9,10]. A potentially more productive approach to 2-alkenyl-2*H*-indazole targets begins with alkylation of indazole with α-bromocarbonyl compounds (path (c), Scheme 1). The major difficulty associated with this process is that *N*-1 thermodynamic alkylation products predominate in reactions run under most basic conditions [18]. Fortunately, the results of recent studies in this area show that 2*H*-indazoles are generated through gallium/aluminium- and aluminium-mediated direct regioselective alkylation reactions of an indazole with α-bromocarbonyl compounds [19]. Below, we describe observations made in an investigation in which this new alkylation procedure was applied to the synthesis of a variety of 2-alkenyl-2*H*-indazoles.

## 2. Results and Discussion

**Figure 1:**
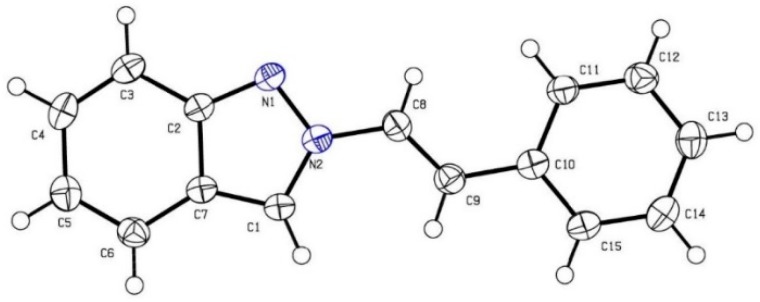
ORTEP plot of X-ray crystallographic data for **2a**.

One approach to the synthesis of 2-alkenyl-2*H*-indazoles **2** from ketones **3a**–**j**, produced by regioselective alkylation reactions of indazoles with α-bromocarbonyl compounds, is based on a reduction-elimination strategy. Specifically, treatment of **3a**–**j** with sodium borohydride generates alcohols, which are then transformed to either chloride or mesylate derivatives to facilitate elimination to form alkenes. The results in which this sequence is applied to selected indazole-ketones are displayed in Table 1. Conversion of the intermediate alcohols to the corresponding chlorides was accomplished by using phosphoryl chloride. Under these conditions, minor amounts of elimination products were formed along with the chloride intermediates. Treatment of these mixtures with DBU led to clean formation of the target 2-alkenyl-2*H*-indazoles **2**. However, elimination product **2e** was formed directly when the corresponding alcohol was treated with phosphoryl chloride. A decomposition process occurred in the chlorination reaction with alcohol intermediate indazole bearing nitro substituent (Table 1, entry 9). The alcohols to alkene transformations can also be accomplished by first forming the mesylate derivatives followed by DBU promoted elimination. Overall, the route B predominated in terms of functional group compatibility and yield. The elimination reactions were observed to generate either *E*-regioisomers exclusively (Table 1, entries 1, 3–6, 8 and 10) or mixtures of *E* and *Z*-isomers (Table 1, entries 2, 7 and 9). The *E* double bond geometry of **2a** was unambiguously assigned by using X-ray crystallography (Figure 1) [20]. The detailed X-ray crystallographic data for **2a** are provided in the Supplementary Materials.

**Scheme 2:**
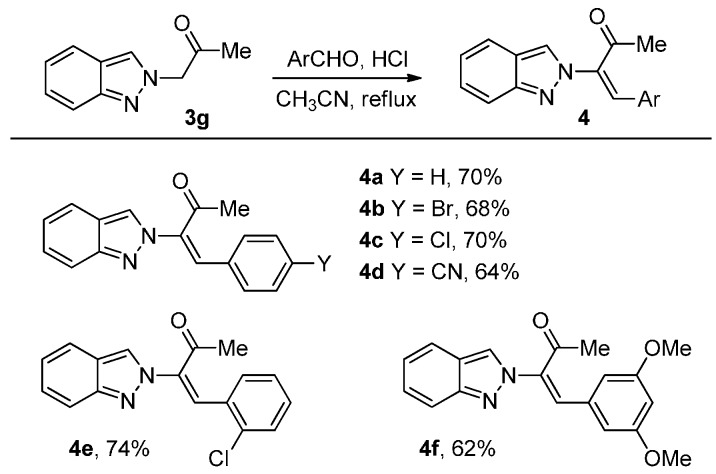
Aldol condensation reactions of **3g** with aldehydes.

**Scheme 3:**
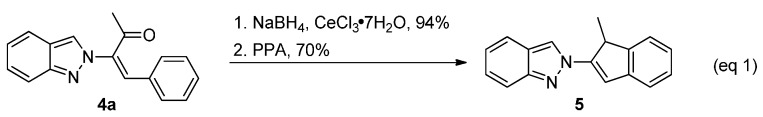
Formation of the indene-linked 2*H*-indazole.

The functionality in the side chain of indazole-ketones **3** is amenable to conversion to α-branched α,β-unsaturated ketones, which have potential as anticancer drugs [21]. As expected, the α-(2*H*-indazol-2-yl)-enones **4a**–**f** can be readily prepared by aldol condensation reactions between ketone **3g** and benzaldehyde derivatives (Scheme 2). Moreover, the indene-linked 2*H*-indazole **5** can be prepared through reduction of the ketone moiety in **4a** followed by polyphosphoric acid (PPA)-promoted intramolecular electrophilic aromatic substitution (Scheme 3, Equation (1)) [22].

**Scheme 4:**
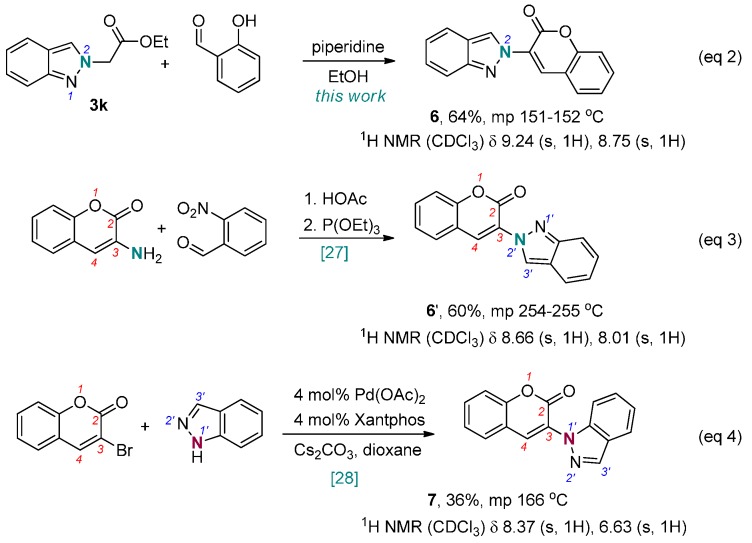
Synthesis of the coumarin-substituted 2*H*-indazoles.

Recently, 2-aryl-2*H*-indazoles have been identified as a new class of fluorescent compounds [15]. In addition, coumarin derivatives have been widely studied and utilized as fluorescent probes in living cells [23,24,25,26]. We envisioned that coumarin-substituted 2*H*-indazoles **6** (Scheme 4, Equation (2)) possessing a planar, rigid π-conjugated system might display interesting luminescent behavior. In studies pursuing this proposal we observed that coumarin-indazole **6** can be generated by condensation reaction of the indazole-ester **3k** with salicylaldehyde (Equation (2)).

**Figure 2:**
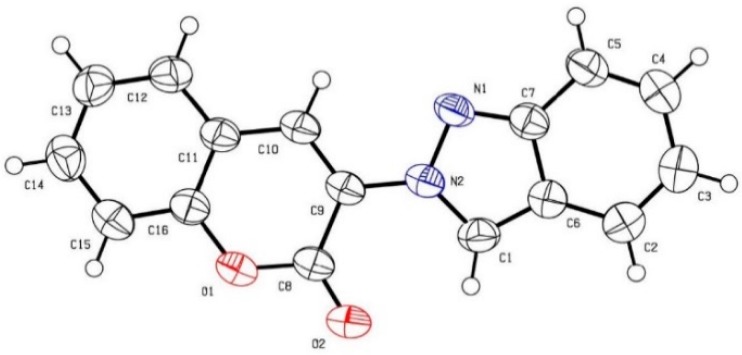
ORTEP plot of the crystallographic data for **6**.

It should be noted that the synthesis of coumarin-indazole **6** was carried out previously through a route that begins with reaction of 3-aminocoumarin with *o*-nitrobenzaldehyde (Scheme 4, Equation (3)) [27]. In addition, coumarin derived *N*1-indazole **7** was synthesized previously by palladium catalyzed coupling of 3-bromo-coumarin with indazole (Scheme 4, Equation (4)) [28]. Interestingly, the NMR spectroscopic data of our synthetic **6** are totally different from those of the substance prepared earlier as well as those of **7**. To gain support for the structural assignment we have made to **6**, X-ray crystallographic analysis was performed (Figure 2) [29]. The conflicting observations uncovered in this effort lend credence to our earlier statement in the Introduction section that “conventional cyclization reactions to prepare members of the 2*H*-indazole family from 2-nitrobenzaldehyde and 2-azidobenzaldehyde are not applicable to the construction of 2-alkenyl-2*H*-indazoles”. Finally, we determined the fluorescence properties of the new compounds prepared in this effort (Table 2). As can be seen in Table 2, the planar, rigid π-conjugated system 2*H*-indazoles **2a** and **5** display strong fluorescence and **6** possesses a large red stark shift.

## 3. Experimental Section

### 3.1. General Information

All commercially available chemicals were used without further purification. TLC analyses were run on glass TLC plates (Silica gel 60 F254) and were visualized using UV and a solution of phosphomolybdic acid in ethanol (5 wt %) or *p*-anisaldehyde stain. Flash chromatography was performed using silica gel (70–230 mesh). ^1^H-NMR spectra were recorded on a 300 MHz spectrometer (Bruker AV-300, Bruker BioSpin GmbH, Karlsruhe, Germany). ^13^C-NMR spectra were recorded at 75 MHz with complete proton decoupling spectrometer. Chemical shifts are reported relative to CHCl_3_ [δ_H_ 7.24, δ_C_ (central line) 77.0]. Mass spectra were recorded under electron impact ionization (EI) conditions, and high-resolution mass spectra were recorded by electron impact ionization with magnetic sector.

### 3.2. Synthesis

#### 3.2.1. General Procedure for the Synthesis of 2-Alkenyl-2*H*-indazoles **2** (Route A)

To a cold solution (ice bath 0–5 °C) of ketone **3** (0.5 mmol) in MeOH/CH_2_Cl_2_ (1/1, 10 mL) NaBH_4_ (0.5 mmol, 1.0 equiv.) was added slowly. The resulting mixture was warmed to ambient temperature (ice bath removal). Reaction was monitored by TLC until no starting material was observed and normally the reaction was stirred at ambient temperature for 2 h. The reaction was then cooled to 0–5 °C and quenched with 2 N HCl (10 mL). CH_2_Cl_2_ (10 mL) was added and the mixture was transferred to a separatory funnel. The aqueous layer was back extracted with CH_2_Cl_2_ (10 mL × 2). The combined organic layers were dried over Na_2_SO_4_, filtered, and concentrated on a rotary evaporator. The residue was dissolved in POCl_3_ (0.5 mL) and the mixture was heated to reflux (110 °C) under N_2_ overnight. The resulting mixture was cooled to 0–5 °C and sat. NaHCO_3_ was added slowly until no bubble was observed. EtOAc (10 mL) was added and the mixture was transferred to a separatory funnel. The aqueous layer was back extracted with EtOAc (10 mL × 2). The combined organic layers were dried over Na_2_SO_4_, filtered through a silica-gel pad (d × l, 3 cm × 3 cm) and concentrated in a rotary evaporator. The residue was dissolved in DBU (1 mL) and the mixture was heated to 90 °C under N_2_ overnight. The mixture was cooled to ambient temperature. EtOAc (10 mL) and H_2_O (10 mL) were added and the mixture was transferred to a separatory funnel. The aqueous layer was back extracted with EtOAc (10 mL × 2). The combined organic layers were dried over Na_2_SO_4_, filtered, and concentrated in a rotary evaporator. The residue was purified by silica gel chromatography using EtOAc/hexanes (1/9) as eluent to give the product **2**.

#### 3.2.2. General Procedure for the Synthesis of 2-Alkenyl-2*H*-indazoles **2** (Route B)

To a cold solution (ice bath 0–5 °C) of ketone **3** (0.5 mmol) in MeOH/CH_2_Cl_2_ (1/1, 10 mL) was added NaBH_4_ (0.5 mmol, 1.0 equiv.) slowly. The resulting mixture was warmed to ambient temperature (ice bath removal). Reaction was monitored by TLC until no starting material was observed and normally the reaction was stirred at ambient temperature for 2 h. The reaction was then cooled to 0–5 °C and quenched with 2 *N* HCl (10 mL). CH_2_Cl_2_ (10 mL) was added and the mixture was transferred to a separatory funnel. The aqueous layer was back extracted with CH_2_Cl_2_ (10 mL × 2). The combined organic layers were dried over Na_2_SO_4_, filtered, and concentrated in a rotary evaporator. The residue was dissolved in CH_2_Cl_2_ (5 mL) and the mixture was cooled 0–5 °C (ice bath) under N_2_. Et_3_N (0.25 mL, 1.8 mmol) was added followed by MsCl (0.07 mL, 0.9 mmol). The resulting mixture was warmed to ambient temperature (ice bath removal). Reaction was monitored by TLC until no starting material was observed and normally the reaction was stirred at ambient temperature for 2 h. The resulting mixture was cooled to 0–5 °C and sat. NaHCO_3_ (5 mL) was added slowly. The mixture was transferred to a separatory funnel. The aqueous layer was back extracted with CH_2_Cl_2_ (10 mL × 2). The combined organic layers were dried over Na_2_SO_4_, filtered and concentrated in a rotary evaporator. The residue was dissolved in DBU (0.6 mL) and the mixture was heated to 90 °C under N_2_ overnight. The mixture was cooled to ambient temperature. EtOAc (10 mL) and H_2_O (10 mL) were added and the mixture was transferred to a separatory funnel. The aqueous layer was back extracted with EtOAc (10 mL × 2). The combined organic layers were dried over Na_2_SO_4_, filtered, and concentrated in a rotary evaporator. The residue was purified by silica gel chromatography using EtOAc/hexanes (1/9) as eluent to give the product **2**.

*(E)-2-Styryl-2H-indazole* (**2a**): Following the general procedure, the title compound was obtained (Route A: 72 mg, 65%, Route B: 99 mg, 90%). A yellow solid, mp 131–133 °C; TLC (EtOAc/hexanes (1:4)) *R_f_* = 0.56; ^1^H-NMR (CDCl_3_) δ 7.07 (t, *J* = 7.8 Hz, 1 H), 7.25–7.37 (m, 4H), 7.43–7.48 (m, 3H), 7.58–7.65 (m, 2H), 7.75 (d, *J* = 7.8 Hz, 1H), 7.97 (s, 1H); ^13^C-NMR (CDCl_3_) δ 117.2 (CH), 120.1 (CH), 120.9 (CH), 121.8 (CH), 122.0 (C), 122.2 (CH), 126.2 (CH), 126.4 (CH × 2), 127.0 (CH), 128.0 (CH), 128.7 (CH × 2), 134.1 (C), 149.4 (C); IR (neat) 3131, 2950, 1645 cm^−1^; EI-MS *m/z* (rel intensity) 220 ([M]^+^, 56), 219 (100), 129 (97), 77 (22); HRMS [M]^+^ calcd for C_15_H_12_N_2_: 220.1000, found 220.1004.

*2-(4-Bromostyryl)-2H-indazole* (**2b**): Following the general procedure, the title compound was obtained (Route A: 78 mg, 52%, Route B: 147 mg, 98%). A yellow solid, mp 154–156 °C; TLC (EtOAc/hexanes (1:4)) *R_f_* = 0.50; ^1^H-NMR (CDCl_3_) δ 7.08 (dd, *J* = 7.8, 6.6 Hz, 1H), 7.28–7.41 (m, 3H), 7.46–7.51 (m, 3H), 7.62–7.75 (m, 3H), 8.10 (s, 0.8H), 8.13 (s, 0.1H); ^13^C-NMR (CDCl_3_) δ 117.3, 119.8, 120.1, 121.1, 121.9, 122.0, 122.1, 122.4, 122.5, 126.5, 126.7, 127.1, 127.3, 127.9, 128.1, 128.8, 131.9, 133.2, 134.2, 149.6; IR (neat) 3062, 2950, 1653 cm^−1^; EI-MS *m*/*z* (rel intensity) 300 ([M + 2]^+^, 71), 299 (100), 298 ([M]^+^, 73), 118 (97); HRMS [M]^+^ calcd for C_15_H_11_BrN_2_: 298.0106, found 298.0101.

*(E)-2-(4-Chlorostyryl)-2H-indazole* (**2c**): Following the general procedure, the title compound was obtained (Route A: 61 mg, 48%, Route B: 96 mg, 75%). A yellow solid, mp 150–151 °C; TLC (EtOAc/hexanes (1:4)) *R_f_* = 0.50; ^1^H-NMR (CDCl_3_) δ 7.06 (dd, *J* = 8.1, 6.9 Hz, 1H), 7.26–7.40 (m, 6H), 7.55–7.59 (m, 2H), 7.71 (d, *J* = 9.0 Hz, 1H), 7.98 (s, 1H); ^13^C-NMR (CDCl_3_) δ 117.3 (CH), 119.7 (CH), 120.1 (CH), 122.0 (CH), 122.1 (C), 122.4 (CH), 126.6 (CH), 127.3 (CH), 127.5 (CH × 2), 128.9 (CH × 2), 132.7 (C), 133.7 (C), 149.6 (C); IR (neat) 3031, 2950, 1393 cm^−1^; EI-MS *m*/*z* (rel intensity) 256 ([M + 2]^+^, 25), 254 ([M]^+^, 81), 253 (100), 118 (80); HRMS [M]^+^ calcd for C_15_H_11_ClN_2_: 254.0611, found 254.0617.

*(E)-2-(4-Methylstyryl)-2H-indazole* (**2d**): Following the general procedure, the title compound was obtained (Route A: 74 mg, 63%, Route B: 93 mg, 79%). A white solid, mp 149–151 °C; TLC (EtOAc/hexanes (1:4)) *R_f_* = 0.60; ^1^H-NMR (CDCl_3_) δ 2.33 (s, 3H), 7.06 (t, *J* = 7.8 Hz, 1H), 7.15 (d, *J* = 7.8 Hz, 2H), 7.23–7.45 (m, 4H), 7.58–7.63 (m, 2H), 7.74 (d, *J* = 8.7 Hz, 1H), 7.98 (s, 1H); ^13^C-NMR (CDCl_3_) δ 21.1 (CH_3_), 117.2 (CH), 120.1 (CH), 121.0 (CH), 121.6 (CH), 122.1 (C), 122.2 (CH), 125.6 (CH), 126.3 (CH × 2), 126.9 (CH), 129.4 (CH × 2), 131.3 (C), 138.1 (C), 149.4 (C); IR (neat) 3121, 1602, 1367 cm^−1^; EI-MS *m*/*z* (rel intensity) 234 ([M]^+^, 71), 233 (100), 218 (14), 118 (27); HRMS [M]^+^ calcd for C_16_H_14_N_2_: 234.1157, found 234.1159.

*(E)-2-(4-Methoxystyryl)-2H-indazole* (**2e**): Following the general procedure, the title compound was obtained (Route A: 101 mg, 81%, Route B: 109 mg, 87%). A white solid, mp 137–139 °C; TLC (EtOAc/hexanes (1:4)) *R_f_* = 0.45; ^1^H-NMR (CDCl_3_) δ 3.78 (s, 3H), 6.88 (d, *J* = 8.7 Hz, 2H), 7.06 (t, *J* = 8.7 Hz, 1H), 7.26 (t, *J* = 8.7 Hz, 1H), 7.31–7.43 (m, 3H), 7.55 (d, *J* = 14.7 Hz, 1H), 7.60 (d, *J* = 8.7 Hz, 1H), 7.72 (d, *J* = 8.7 Hz, 1H), 8.00 (s, 1H); ^13^C-NMR (CDCl_3_) δ 55.1 (CH_3_), 114.2 (CH × 2), 117.2 (CH), 120.0 (CH), 120.8 (CH), 121.5 (CH), 122.1 (C), 122.2 (CH), 124.6 (CH), 126.7 (C), 126.9 (CH), 127.8 (CH × 2), 149.3 (C), 159.6 (C); IR (neat) 3120, 1609, 1517 cm^−1^; EI-MS *m*/*z* (rel intensity) 250 ([M]^+^, 100), 249 (58), 132 (93), 118 (19); HRMS [M]^+^ calcd for C_16_H_14_N_2_O: 250.1106, found 250.1104.

*(E)-2-(2-(Naphthalen-2-yl)vinyl)-2H-indazole* (**2f**): Following the general procedure, the title compound was obtained (Route A: 85 mg, 63%, Route B: 115 mg, 85%). A yellow solid, mp 195–196 °C; TLC (EtOAc/hexanes (1:4)) *R_f_* = 0.52; ^1^H-NMR (DMSO-*d*_6_) δ 7.08 (t, *J* = 7.8 Hz, 1H), 7.31 (t, *J* = 7.8 Hz, 1H), 7.49–7.55 (m, 2H), 7.67–7.77 (m, 3H), 7.88–7.96 (m, 4H), 8.08 (s, 1H), 8.41 (d, *J* = 14.1 Hz, 1 H), 8.68 (s, 1H); ^13^C-NMR (DMSO-*d*_6_) δ 117.0 (CH), 120.3 (CH), 120.9 (CH), 121.9 (CH × 2), 123.4 (CH), 123.7 (CH), 126.2 (CH), 126.6 (CH), 126.8 (CH), 127.0 (CH), 127.6 (CH), 127.7 (C), 127.8 (CH), 128.4 (CH), 132.2 (C), 132.6 (C), 133.2 (C) 148.9 (C); IR (neat) 3062, 2950, 1626 cm^−1^; EI-MS *m*/*z* (rel intensity) 270 ([M]^+^, 93), 269 (100), 152 (90), 118 (11); HRMS [M]^+^ calcd for C_19_H_14_N_2_: 270.1157, found 270.1150.

*2-(Prop-1-en-1-yl)-2H-indazole* (**2g**): Following the general procedure, the title compound was obtained (Route A: 57 mg, 72%, Route B: 66 mg, 84%). A yellow oil; TLC (EtOAc/hexanes (1:2)) *R_f_* = 0.58; ^1^H-NMR (CDCl_3_) δ 1.85 (dd, *J* = 6.9, 1.8 Hz, 2.60H), 2.02 (dd, *J* = 6.9, 1.5 Hz, 0.40H), 5.60–5.62 (m, 0.13H), 6.39–6.99 (m, 0.87H), 7.00–7.07 (m, 2H), 7.21–7.28 (m, 1H), 7.55–7.72 (m, 2H), 7.89 (s, 0.87H), 7.96 (s, 0.13H), 8.65 (s, 0.87H), 8.70 (s, 0.13H); ^13^C-NMR (CDCl_3_) δ 12.9, 14.8, 117.3, 117.7, 120.0, 120.5, 121.8, 121.9, 126.5, 128.3, 149.0; IR (neat) 3113, 2933, 1624 cm^−1^; EI-MS *m*/*z* (rel intensity) 158 ([M]^+^, 46), 157 (28), 131 (100), 118 (12); HRMS [M]^+^ calcd for C_10_H_10_N_2_: 158.0844, found 158.0843.

*(E)-2-(3,3-Dimethylbut-1-en-1-yl)-2H-indazole* (**2h**): Following the general procedure, the title compound was obtained (Route A: 24 mg, 24%, Route B: 70 mg, 70%). An oil; TLC (EtOAc/hexanes (1:2)) *R_f_* = 0.70; ^1^H-NMR (CDCl_3_) δ 1.18 (s, 9H), 6.59 (d, *J* = 14.4 Hz, 1H), 7.01 (d, *J* = 14.4 Hz, 1H), 7.05 (d, *J* = 8.7 Hz, 1H), 7.27 (d, *J* = 8.7 Hz, 1H), 7.61 (d, *J* = 8.7 Hz, 1H), 7.68 (d, *J* = 8.7 Hz, 1H), 8.00 (s, 1H); ^13^C-NMR (CDCl_3_) δ 25.6 (CH_3_ × 3), 32.3 (C), 117.3 (CH), 120.0 (CH), 121.0 (CH), 121.8 (C), 122.0 (CH), 124.5 (CH), 126.5 (CH), 133.4 (CH), 149.1 (C); IR (neat) 3062, 2950, 1633 cm^−1^; EI-MS *m*/*z* (rel intensity) 200 ([M]^+^, 74), 185 (100), 131 (57), 118 (58); HRMS [M]^+^ calcd for C_13_H_16_N_2_: 200.1313, found 200.1315.

*6-Nitro-2-(prop-1-en-1-yl)-2H-indazole* (**2i**): Following the general procedure, the title compound was obtained (Route B: 81 mg, 80%). A green solid, mp 103–105 °C; TLC (EtOAc/hexanes (1:4)) *R_f_* = 0.20; ^1^H-NMR (acetone-*d*_6_) δ 1.92 (d, *J* = 6.9 Hz, 2.85H), 2.15 (d, *J* = 6.9 Hz, 0.15H), 5.78–5.88 (m, 0.05H), 6.68–6.80 (m, 0.95H), 7.33–7.40 (m, 1H), 7.78–7.91 (m, 2H), 8.54 (s, 1.9H), 8.55 (s, 0.1H); ^13^C-NMR (acetone-*d*_6_) δ 15.0 (CH_3_), 115.7 (CH), 116.2 (CH), 121.1 (CH), 123.2 (CH), 123.8 (CH), 125.3 (C), 129.5 (CH), 147.7 (C), 147.8 (C); IR (neat) 3051, 1514, 1342 cm^−1^; EI-MS *m*/*z* (rel intensity) 203 ([M]^+^, 100), 176 (95), 156 (27), 130 (47); HRMS [M]^+^ calcd for C_10_H_9_N_3_O_2_: 203.0695, found 203.0699.

*(E)-6-Bromo-2-styryl-2H-indazole* (**2j**): Following the general procedure, the title compound was obtained (Route A: 112 mg, 75%, Route B: 120 mg, 80%). A yellow solid, mp 118–120 °C; TLC (EtOAc/hexanes (1:4)) *R_f_* = 0.50; ^1^H-NMR (CDCl_3_) δ 7.15 (dd, *J* = 9.0, 1.5 Hz, 1H), 7.28–7.40 (m, 3H), 7.44–7.52 (m, 4H), 7.67 (d, *J* = 14.4 Hz, 1H), 7.89 (s, 1H), 8.09 (s, 1H); ^13^C-NMR (CDCl_3_) δ 119.7 (CH), 120.6 (C), 121.1 (C), 121.5 (CH), 121.7 (CH), 122.2 (CH), 126.0 (CH), 126.1 (CH × 2), 126.5 (CH), 128.3 (CH), 128.8 (CH × 2), 133.9 (C), 150.0 (C); IR (neat) 3062, 2950, 1625 cm^−1^; EI-MS *m*/*z* (rel intensity) 300 ([M + 2]^+^, 56), 299 (100), 298 ([M]^+^, 61), 77 (32); HRMS [M]^+^ calcd for C_15_H_11_BrN_2_: 298.0106, found 298.0109.

#### 3.2.3. General procedure for the synthesis of 2-alkenyl-2*H*-indazoles **4**: 

A mixture of ketone **3g** (1.0 mmol), aldehyde (1.5 mmol), and conc. HCl (3 drops) in CH_3_CN (1 mL) was heated to reflux under nitrogen. Reaction was monitored by TLC until no starting material was observed and normally the reaction was stirred under reflux for 4 h. The reaction was then cooled to ambient temperature and quenched with sat. NaHCO_3_ until pH was 7. CH_2_Cl_2_ (10 mL) was added and the mixture was transferred to a separatory funnel. The aqueous layer was back extracted with CH_2_Cl_2_ (10 mL × 2). The combined organic layers were dried over MgSO_4_, filtered, and concentrated in a rotary evaporator. The residue was purified by silica gel chromatography using EtOAc/hexanes (1/10) as eluent to give the product **4**.

*(E)-3-(2H-Indazol-2-yl)-4-phenylbut-3-en-2-one* (**4a**): Following the general procedure, the title compound was obtained (184 mg, 70%). A yellow oil; TLC (EtOAc/hexanes (1:2)) *R_f_* = 0.35; ^1^H-NMR (acetone-*d*_6_) δ 2.20 (s, 3H), 6.83 (d, *J* = 7.5 Hz, 2H), 7.06–7.16 (m, 3H), 7.25–7.33 (m, 2H), 7.69–7.73 (m, 2H), 7.95 (s, 1H), 8.22 (s, 1H); ^13^C-NMR (acetone-*d*_6_) δ 26.3 (CH_3_), 119.2 (CH), 122.2 (CH), 123.3 (CH), 123.9 (C), 126.9 (CH), 127.8 (CH), 130.0 (CH × 2), 131.7 (CH × 2), 132.2 (CH), 133.1 (C), 138.2 (C), 138.3 (CH), 151.0 (C), 194.8 (C); IR (neat) 3062, 1683, 1610 cm^−1^; EI-MS *m*/*z* (rel intensity) 262 ([M]^+^, 46), 261 (100), 219 (34), 118 (41); HRMS [M]^+^ calcd for C_17_H_14_N_2_O: 262.1106, found 262.1099.

*(E)-4-(4-Bromophenyl)-3-(2H-indazol-2-yl)but-3-en-2-one* (**4b**): Following the general procedure, the title compound was obtained (232 mg, 68%). A yellow oil; TLC (EtOAc/hexanes (1:2)) *R_f_* = 0.38; ^1^H-NMR (CDCl_3_) δ 2.12 (s, 3H), 6.55 (d, *J* = 8.7 Hz, 2H), 7.09–7.14 (m, 1H), 7.24 (d, *J* = 8.7 Hz, 2H), 7.30–7.36 (m, 1H), 7.64 (d, *J* = 8.7 Hz, 1H), 7.74–7.80 (m, 2H), 7.90 (s, 1H); ^13^C-NMR (CDCl_3_) δ 25.6 (CH_3_), 118.0 (CH), 120.4 (CH), 122.4 (C), 122.6 (CH), 124.6 (CH), 125.7 (C), 127.0 (CH), 129.9 (C), 131.6 (CH × 2), 132.0 (CH × 2), 135.7 (CH), 136.4 (C), 149.8 (C), 193.9 (C); IR (neat) 3062, 1674, 1619 cm^−1^; EI-MS *m/z* (rel intensity) 342 ([M + 2]^+^, 49), 341 (100), 340 ([M]^+^, 49), 118 (88); HRMS [M]^+^ calcd for C_17_H_13_BrN_2_O: 340.0211, found 340.0214.

*(E)-4-(4-Chlorophenyl)-3-(2H-indazol-2-yl)but-3-en-2-one* (**4c**): Following the general procedure, the title compound was obtained (208 mg, 70%). A yellow oil; TLC (EtOAc/hexanes (1:2)) *R_f_* = 0.38; ^1^H-NMR (CDCl_3_) δ 2.12 (s, 3 H), 6.62 (d, *J* = 8.7 Hz, 2H), 7.05–7.14 (m, 3H), 7.30–7.36 (m, 2H), 7.64 (d, *J* = 8.7 Hz, 1H), 7.75–7.80 (m, 2H), 7.90 (s, 1H); ^13^C-NMR (CDCl_3_) δ 25.6 (CH_3_), 118.1 (CH), 120.4 (CH), 122.4 (C), 122.6 (CH), 124.6 (CH), 127.0 (CH), 129.1 (CH × 2), 129.5 (C), 131.5 (CH × 2), 135.7 (CH), 136.4 (C), 137.2 (C), 149.9 (C), 194.0 (C); IR (neat) 3062, 1683, 1491 cm^−1^; EI-MS *m/z* (rel intensity) 298 ([M + 2]^+^, 18), 296 ([M]^+^, 51), 295 (100), 118 (64); HRMS [M]^+^ calcd for C_17_H_13_ClN_2_O: 296.0716, found 296.0708.

*(E)-4-(2-(2H-Indazol-2-yl)-3-oxobut-1-en-1-yl)benzonitrile* (**4d**): Following the general procedure, the title compound was obtained (184 mg, 64%). A yellow oil; TLC (EtOAc/hexanes (1:2)) *R_f_* = 0.20; ^1^H-NMR (CDCl_3_) δ 2.16 (s, 3H), 6.68 (d, *J* = 8.7 Hz, 2H), 7.13 (t, *J* = 8.1 Hz, 1H), 7.32–7.41 (m, 3H), 7.63 (d, *J* = 8.1 Hz, 1H), 7.74 (d, *J* = 8.1 Hz, 1H), 7.78 (s, 1H), 7.88 (s, 1H); ^13^C-NMR (CDCl_3_) δ 25.8 (CH_3_), 113.8 (C), 117.8 (C), 118.0 (CH), 120.4 (CH), 122.4 (C), 122.9 (CH), 124.7 (CH), 127.3 (CH), 130.4 (CH × 2), 132.3 (CH × 2), 134.3 (CH), 135.4 (C), 138.1 (C), 149.9 (C), 193.9 (C); IR (neat) 3064, 1677, 1631 cm^−1^; EI-MS *m*/*z* (rel intensity) 287 ([M]^+^, 55), 286 (100), 244 (25), 118 (30); HRMS [M]^+^ calcd for C_18_H_13_N_3_O: 287.1059, found 287.1049.

*(E)-4-(2-Chlorophenyl)-3-(2H-indazol-2-yl)but-3-en-2-one* (**4e**): Following the general procedure, the title compound was obtained (220 mg, 74%). A yellow oil; TLC (EtOAc/hexanes (1:2)) *R_f_* = 0.50; ^1^H-NMR (CDCl_3_) δ 2.21 (s, 3H), 6.30 (d, *J* = 8.1 Hz, 1H), 6.78 (d, *J* = 8.1 Hz, 1H), 7.03–7.16 (m, 2H), 7.26–7.37 (m, 2H), 7.55 (d, *J* = 8.1 Hz, 1H), 7.75 (d, *J* = 8.1 Hz, 1H), 7.79 (s, 1H), 8.10 (s, 1H); ^13^C-NMR (CDCl_3_) δ 25.9 (CH_3_), 118.0 (CH), 120.4 (CH), 122.1 (C), 122.4 (CH), 125.1 (CH), 126.9 (CH), 127.0 (CH), 129.4 (CH), 129.6 (CH), 129.9 (C), 131.4 (CH), 132.5 (CH), 135.7 (C), 137.9 (C), 149.7 (C), 193.8 (C); IR (neat) 3053, 1693, 1624 cm^−1^; EI-MS *m*/*z* (rel intensity) 296 ([M]^+^, 3), 262 (18), 261 (100), 118 (9); HRMS [M]^+^ calcd for C_17_H_13_ClN_2_O: 296.0716, found 296.0718.

*(E)-4-(3,5-Dimethoxyphenyl)-3-(2H-indazol-2-yl)but-3-en-2-one* (**4f**): Following the general procedure, the title compound was obtained (200 mg, 62%). A brown solid, mp 94–96 °C; TLC (EtOAc/hexanes (1:2)) *R_f_* = 0.35; ^1^H-NMR (CDCl_3_) δ 2.19 (s, 3H), 3.22 (s, 6H), 5.72 (d, *J* = 2.4 Hz, 2H), 6.30 (t, *J* = 2.4 Hz, 1H), 7.04–7.09 (m, 1H), 7.25–7.30 (m, 1H), 7.60 (d, *J* = 8.1 Hz, 1H), 7.72–7.75 (m, 2 H), 7.90 (s, 1H); ^13^C- NMR (CDCl_3_) δ 25.7 (CH_3_), 54.6 (CH_3_ × 2), 104.4 (CH), 107.4 (CH × 2), 117.7 (CH), 120.3 (CH), 122.4 (C), 122.5 (CH), 125.1 (CH), 126.9 (CH), 132.3 (C), 136.2 (C), 137.9 (CH), 149.7 (C), 160.4 (C × 2), 194.0 (C); IR (neat) 3120, 1697, 1583 cm^−1^; EI-MS *m*/*z* (rel intensity) 322 ([M]^+^, 78), 321 (100), 279 (21), 189 (32); HRMS [M]^+^ calcd for C_19_H_18_N_2_O_3_: 322.1317, found 322.1318.

*2-(1-Methyl-1H-inden-2-yl)-2H-indazole* (**5**). To a solution of ketone **4a** (184 mg, 0.7 mmol) in MeOH (7 mL) was added CeCl_3_–7H_2_O (135 mg, 0.36 mmol) followed by NaBH_4_ (28 mg, 0.7 mmol, 1.0 equiv.) at ambient temperature. The reaction was stirred at ambient temperature for 2 h and then quenched with H_2_O (10 mL). CH_2_Cl_2_ (10 mL) was added and the mixture was transferred to a separatory funnel. The aqueous layer was back extracted with CH_2_Cl_2_ (10 mL × 2). The combined organic layers were dried over MgSO_4_, filtered, and concentrated in a rotary evaporator. The residue (174 mg) was dissolved in DCE (2.4 mL) and PPA (0.66 mL) was added. The mixture was heated to 90 °C under N_2_ for 2 h. The resulting mixture was cooled to 0–5 °C and sat. NaHCO_3_ was added slowly until pH was 7. CH_2_Cl_2_ (10 mL) was added and the mixture was transferred to a separatory funnel. The aqueous layer was back extracted with CH_2_Cl_2_ (10 mL × 2). The combined organic layers were dried over MgSO_4_, filtered, and concentrated in a rotary evaporator. The residue was purified by silica gel chromatography using EtOAc/hexanes (1/200) as eluent to give the product **5**. (121 mg, 70%). A yellow solid, mp 74–75 °C; TLC (EtOAc/hexanes (1:2)) *R_f_* = 0.63; ^1^H-NMR (CDCl_3_) δ 1.52 (d, *J* = 7.5 Hz, 3H), 4.23 (q, *J* = 7.5 Hz, 1H), 7.10 (t, *J* = 7.5 Hz, 2H), 7.23–7.45 (m, 5H), 7.66 (d, *J* = 8.7 Hz, 1H), 7.80 (d, *J* = 8.7 Hz, 1H), 8.20 (s, 1H); ^13^C-NMR (CDCl_3_) δ 16.9 (CH_3_), 43.2 (CH), 117.4 (CH), 117.6 (CH), 120.1 (CH), 121.0 (CH), 121.5 (CH), 122.2 (C), 122.4 (CH), 122.7 (CH), 125.4 (CH), 126.9 (CH), 127.0 (CH), 141.0 (C), 145.9 (C), 149.6 (C), 149.7 (C); IR (neat) 3062, 1634, 1486 cm^−1^; EI-MS *m*/*z* (rel intensity) 246 ([M]^+^, 100), 245 (92), 231 (34), 128 (45); HRMS [M]^+^ calcd for C_17_H_14_N_2_: 246.1157, found 246.1155.

*3-(2H-Indazol-2-yl)-2H-chromen-2-one* (**6**). A mixture of ethyl 2-(2*H*-indazol-2-yl)acetate (204 mg, 1.0 mmol), 2-hydroxybenzaldehyde (122 mg, 1.0 mmol), and piperidine (43 mg, 0.5 mmol) in EtOH (1 mL) was heated to reflux. After 15 h, the reaction was then cooled to ambient temperature and quenched with sat. NH_4_Cl until pH was 7. CH_2_Cl_2_ (10 mL) was added and the mixture was transferred to a separatory funnel. The aqueous layer was back extracted with CH_2_Cl_2_ (10 mL × 2). The combined organic layers were dried over MgSO_4_, filtered, and concentrated in a rotary evaporator. The residue was purified by silica gel chromatography using EtOAc/hexanes (1/10) as eluent to give the product **6** (167 mg, 64%). A yellow solid, mp 151–152 °C; TLC (EtOAc/hexanes (1:4)) *R_f_* = 0.45; ^1^H-NMR (DMSO-*d*_6_) δ 7.11 (d, *J* = 7.8 Hz, 1H), 7.35 (t, *J* = 7.8 Hz, 1H), 7.46 (t, *J* = 7.8 Hz, 1H), 7.54 (d, *J* = 7.8 Hz, 1H), 7.67–7.73 (m, 2H), 7.82 (d, *J* = 7.8 Hz, 1H), 8.05 (d, *J* = 7.8 Hz, 1H), 8.87 (s, 1H), 9.17 (s, 1H); ^13^C-NMR (DMSO-*d*_6_) δ 116.1 (CH), 117.0 (CH), 118.6 (C), 121.5 (CH), 122.0 (C), 122.4 (CH), 125.4 (CH), 125.5 (CH), 125.8 (C), 127.9 (CH), 129.5 (CH), 132.5 (CH), 134.1 (CH), 148.6 (C), 152.0 (C), 156.2 (C); IR (neat) 3154, 1722, 1600 cm^−1^; EI-MS *m*/*z* (rel intensity) 262 ([M]^+^, 79), 236 (77), 127 (64), 105 (100); HRMS [M]^+^ calcd for C_16_H_10_N_2_O_2_: 262.0742, found 262.0734.

## 4. Conclusions

In conclusion, the studies described above have led to the development of a method for the synthesis of 2-alkenyl-2*H*-indazoles from α-(2*H*-indazol-2-yl)ketones. The 2-alkenyl-2*H*-indazole products may be potentially useful as fluorescent probes or as anticancer drugs.

## Figures and Tables

**Table 1:** Synthesis of 2-alkenyl-2*H*-indazoles **2** from ketones **3**. 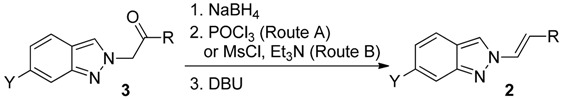

Entry	Product	2; Yield(%) ^a^ from Route A	2; Yield (%) ^a^ from Route B
1	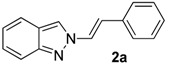	65	90
2	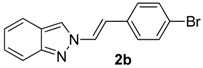	52 (10:1) ^c^	98 (8:1) ^c^
3	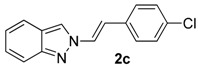	48	75
4	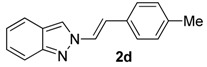	63	79
5	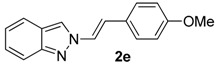	81 ^b^	87
6	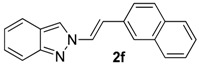	63	85
7	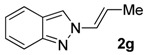	72 (10:1) ^c^	84 (6.5:1) ^c^
8	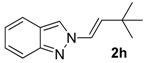	24	70
9	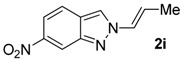	decomposed	80 (20:1) ^c^
10	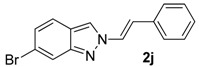	75	80

^a^: Three-step overall yield. ^b^: Two-step overall yield. ^c^: *E*/*Z* ratio was determined by using ^1^H-NMR.

**Table 2:** Fluorescence Properties ^a^.

Compd.	λ_abs_ (nm)	λ_em_ (nm)	ε(M^−1^cm^−1^)	φ
**2a**	322	398, 415	35,400	0.65
**5**	336	402	29,949	0.71
**6**	345	517	24,012	0.01

^a^ Fluorescence measurements made on 10^−5^ M THF solutions of the substances.

## Supplementary Materials

Supplementary materials can be accessed at: http://www.mdpi.com/1420-3049/21/2/238/s1.
