# Supplementary material for: Synthesis of 2-Alkenyl-2H-indazoles from 2-(2-Carbonylmethyl)-2H-indazoles
Source: Molecules. 2016 Feb 19;21(2):238. doi: 10.3390/molecules21020238 (PMC6273450; doi:10.3390/molecules21020238)
Supplement: Supplementary file 1 [file molecules-21-00238-s001.pdf]

Supplementary Materials: Synthesis of 2-Alkenyl-2*H*-indazoles from 2-(2-Carbonylmethyl)-2*H*-indazoles

Mei-Huey Lin *, Kung-Yu Liang, Chang-Hsien Tsai, Yu-Chun Chen, Hung-Chang Hsiao, Yi-Syuan Li, Chung-Hao Chen and Hau-Chun Wu

Table of Contents:

I. X-ray Crystallographic Analyses. Page S1-S7
II. Copies of ^{1}H and ^{13}C NMR spectra. Page S8-S43

I. X-ray Crystallographic Analyses

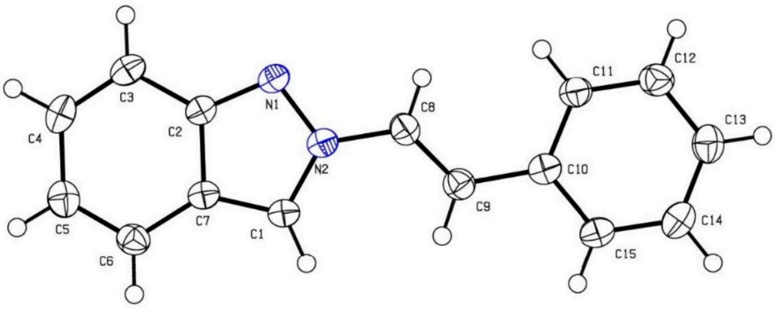

Figure S1. ORTEP plot of X-ray crystallographic data for **2a**.

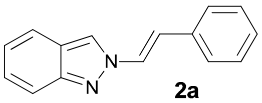

2a

Table S1. Crystal data and structure refinement for **2a** (CCDC: 1442986, LKYIII901).

Identification Code	lkyiii901	
Empirical formula	C15 H20 N2	
Formula weight	228.33	
Temperature	150 K	
Wavelength	0.71073 Å	
Crystal system	Orthorhombic	
Space group	Pca2(1)	
Unit cell dimensions	a = 7.8713(4) Å	α = 90°
	b = 5.7010(3) Å	β = 90°
	c = 25.0463(13) Å	γ = 90°
Volume	1123.93(10) Å^3	
Z	4	
Density (calculated)	1.349 Mg/m^3	
Absorption coefficient	0.080 mm^{-1}	
F(000)	496	
Crystal size	0.20 × 0.09 × 0.01 mm^3	
Theta range for data collection	3.25 to 28.74°	

Index ranges	−10 ≤ h ≤ 10, −7 ≤ k ≤ 7, −33 ≤ l ≤ 33
Reflections collected	15,208
Independent reflections	2922 [R(int) = 0.0899]
Completeness to theta = 28.74°	99.9%
Absorption correction	Semi-empirical from equivalents
Max. and min. transmission	0.9992 and 0.9842
Refinement method	Full-matrix least-squares on F^2
Data/restraints/parameters	2922/1/154
Goodness-of-fit on F^2	0.854
Final R indices [I > 2sigma(I)]	R^1 = 0.0518, wR^2 = 0.1218
R indices (all data)	R^1 = 0.0853, wR^2 = 0.1459
Absolute structure parameter	−2(4)
Largest diff. peak and hole	0.184 and −0.232 e·Å^{-3}

Table S2. Atomic coordinates (×10^4) and equivalent isotropic displacement parameters (Å^2 × 10^3 for LKYIII901. U(eq) is defined as one third of the trace of the orthogonalized U^{ij} tensor.

	x	y	z	U(eq)
C(7)	733(3)	6181(4)	9911(1)	25(1)
C(9)	2586(3)	3665(4)	8390(1)	30(1)
C(10)	3154(3)	3123(4)	7847(1)	28(1)
C(5)	−555(3)	7436(4)	10,719(1)	34(1)
C(8)	2064(3)	5761(4)	8548(1)	30(1)
C(2)	257(3)	8341(4)	9663(1)	24(1)
C(14)	4570(4)	473(5)	7238(1)	35(1)
C(11)	2809(3)	4605(4)	7414(1)	30(1)
C(12)	3351(4)	4036(5)	6906(1)	35(1)
C(13)	4230(3)	1958(5)	6817(1)	36(1)
C(15)	4030(3)	1050(4)	7749(1)	31(1)
C(6)	337(3)	5766(4)	10,452(1)	30(1)
C(3)	−673(3)	10,046(4)	9950(1)	30(1)
C(4)	−1086(3)	9563(5)	10,466(1)	32(1)
N(2)	1502(2)	6299(3)	9069(1)	28(1)
C(1)	1533(3)	4920(4)	9508(1)	27(1)
N(1)	740(3)	8419(3)	9146(1)	27(1)

Table S3. Bond lengths [Å] and angles [°] for LKYIII901 [a].

**C(7)-C(1)**	**1.389(3)**
C(7)-C(6)	1.411(3)
C(7)-C(2)	1.429(3)
C(9)-C(8)	1.324(3)
C(9)-C(10)	1.465(3)
C(10)-C(15)	1.390(3)
C(10)-C(11)	1.401(3)
C(5)-C(6)	1.359(4)
C(5)-C(4)	1.430(4)
C(8)-N(2)	1.409(3)
C(2)-N(1)	1.350(3)
C(2)-C(3)	1.414(3)
C(14)-C(13)	1.378(4)
C(14)-C(15)	1.388(4)
C(11)-C(12)	1.382(4)
C(12)-C(13)	1.390(4)
C(3)-C(4)	1.360(4)

N(2)-C(1)	1.354(3)
N(2)-N(1)	1.363(3)
C(1)-C(7)-C(6)	135.3(2)
C(1)-C(7)-C(2)	104.5(2)
C(6)-C(7)-C(2)	120.2(2)
C(8)-C(9)-C(10)	124.3(2)
C(15)-C(10)-C(11)	118.1(2)
C(15)-C(10)-C(9)	119.7(2)
C(11)-C(10)-C(9)	122.1(2)
C(6)-C(5)-C(4)	121.7(2)
C(9)-C(8)-N(2)	124.8(2)
N(1)-C(2)-C(3)	127.7(2)
N(1)-C(2)-C(7)	111.8(2)
C(3)-C(2)-C(7)	120.5(2)
C(13)-C(14)-C(15)	120.0(2)
C(12)-C(11)-C(10)	120.8(2)
C(11)-C(12)-C(13)	120.1(3)
C(14)-C(13)-C(12)	119.9(3)
C(14)-C(15)-C(10)	121.2(2)
C(5)-C(6)-C(7)	118.0(2)
C(4)-C(3)-C(2)	117.9(2)
C(3)-C(4)-C(5)	121.5(2)
C(1)-N(2)-N(1)	114.0(2)
C(1)-N(2)-C(8)	128.3(2)
N(1)-N(2)-C(8)	117.56(19)
N(2)-C(1)-C(7)	106.4(2)
C(2)-N(1)-N(2)	103.38(18)

[a] Symmetry transformations used to generate equivalent atoms.

Table S4. Anisotropic displacement parameters ($Å^2 \times 10^3$) for LKYIII901. The anisotropic displacement factor exponent takes the form: $-2\pi^2[h^2 a^{*2}U^{11} + ... + 2 h k a^* b^* U^{12}]$.

	**U^{11}**	**U^{22}**	**U^{33}**	**U^{23}**	**U^{13}**	**U^{12}**
C(7)	23(1)	22(1)	30(1)	1(1)	−4(1)	−2(1)
C(9)	30(1)	30(1)	29(1)	0(1)	−3(1)	0(1)
C(10)	25(1)	27(1)	32(1)	0(1)	−3(1)	−1(1)
C(5)	34(1)	35(2)	32(1)	−3(1)	3(1)	−6(1)
C(8)	31(1)	32(1)	26(1)	1(1)	0(1)	0(1)
C(2)	19(1)	24(1)	30(1)	0(1)	−2(1)	−1(1)
C(14)	32(1)	31(1)	42(2)	−7(1)	1(1)	1(1)
C(11)	32(1)	27(1)	32(1)	0(1)	−3(1)	2(1)
C(12)	39(1)	32(1)	33(1)	0(1)	−5(1)	0(1)
C(13)	38(2)	38(1)	33(2)	−7(1)	1(1)	−3(1)
C(15)	32(1)	24(1)	38(1)	2(1)	−1(1)	0(1)
C(6)	33(1)	27(1)	30(1)	2(1)	−1(1)	−4(1)
C(3)	25(1)	24(1)	40(2)	−3(1)	−4(1)	0(1)
C(4)	26(1)	34(1)	38(1)	−11(1)	2(1)	1(1)
N(2)	28(1)	25(1)	30(1)	1(1)	0(1)	3(1)
C(1)	26(1)	24(1)	29(1)	2(1)	−2(1)	2(1)
N(1)	28(1)	24(1)	31(1)	−1(1)	−2(1)	2(1)

Table S5. Hydrogen coordinates (×10⁴) and isotropic displacement parameters (Å² × 10³) for LKYIII901.

	x	y	z	U(eq)
H(9)	2592	2459	8640	36
H(5)	−828	7189	11,076	41
H(8)	2068	6966	8298	36
H(14)	5162	−916	7179	42
H(11)	2207	5988	7470	36
H(12)	3127	5044	6623	42
H(13)	4588	1571	6475	43
H(15)	4257	33	8030	38
H(6)	677	4391	10,621	37
H(3)	−993	11,453	9792	36
H(4)	−1727	10,640	10,659	39
H(1)	1998	3425	9536	32

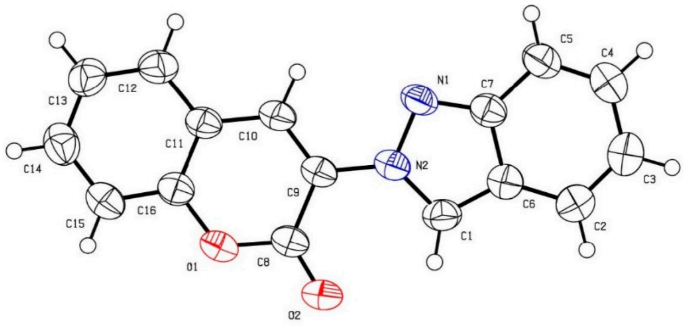

Figure S2. ORTEP plot of the crystallographic data of **6** (CCDC: 1442987).

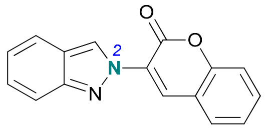

6

Table S6. Crystal data and structure refinement for **6**.

**Identification Code**	**0211**	
Empirical formula	C16 H10 N2 O2	
Formula weight	262.26	
Temperature	296(2) K	
Wavelength	0.71073 Å	
Crystal system	Orthorhombic	
Space group	Pbca	
Unit cell dimensions	a = 13.122(2) Å	α = 90°
	b = 9.5421(17) Å	β = 90°
	c = 19.374(3) Å	γ = 90°
Volume	2425.9(7) $Å^3$	
Z	8	
Density (calculated)	1.442 Mg/m^3	
Absorption coefficient	0.097 mm^{-1}	
F(000)	1096	
Crystal size	0.13 × 0.08 × 0.07 mm^3	
Theta range for data collection	2.10 to 28.28°	

Index ranges	−17 ≤ h ≤ 17, −12 ≤ k ≤ 12, −25 ≤ l ≤ 25
Reflections collected	28,899
Independent reflections	3013 [R(int) = 0.0928]
Completeness to theta = 28.28°	100.0%
Absorption correction	Semi-empirical from equivalents
Max. and min. transmission	0.9932 and 0.9875
Refinement method	Full-matrix least-squares on F^2
Data/restraints/parameters	3013/0/181
Goodness-of-fit on F^2	1.004
Final R indices [I > 2sigma(I)]	R^1 = 0.0494, wR^2 = 0.1071
R indices (all data)	R^1 = 0.1193, wR^2 = 0.1382
Largest diff. peak and hole	0.155 and −0.186 e·Å^{-3}

Table S7. Atomic coordinates (×10^4) and equivalent isotropic displacement parameters (Å^2 × 10^3) for **6**. U(eq) is defined as one third of the trace of the orthogonalized U^{ij} tensor.

	**x**	**y**	**z**	**U(eq)**
O(1)	−410(1)	7923(1)	5030(1)	50(1)
O(2)	−785(1)	9648(2)	5730(1)	60(1)
N(2)	1218(1)	9738(2)	6296(1)	44(1)
N(1)	2236(1)	9847(2)	6427(1)	49(1)
C(3)	2060(2)	12,893(2)	7919(1)	62(1)
C(2)	1207(2)	12,405(2)	7608(1)	58(1)
C(6)	1309(2)	11,346(2)	7103(1)	48(1)
C(1)	645(2)	10,611(2)	6689(1)	51(1)
C(9)	907(1)	8752(2)	5790(1)	42(1)
C(10)	1556(2)	7805(2)	5530(1)	46(1)
C(11)	1248(1)	6868(2)	4996(1)	45(1)
C(12)	1888(2)	5856(2)	4707(1)	54(1)
C(13)	1540(2)	4994(2)	4193(1)	59(1)
C(14)	548(2)	5125(2)	3956(1)	60(1)
C(4)	3033(2)	12,358(2)	7746(1)	62(1)
C(5)	3161(2)	11,354(2)	7257(1)	58(1)
C(7)	2283(1)	10,826(2)	6922(1)	46(1)
C(16)	260(2)	6962(2)	4749(1)	45(1)
C(8)	−140(2)	8839(2)	5540(1)	45(1)
C(15)	−104(2)	6106(2)	4231(1)	54(1)

Table S8. Bond lengths [Å] and angles [°] for **6** [a].

**O(1)-C(8)**	**1.366(2)**
O(1)-C(16)	1.381(2)
O(2)-C(8)	1.204(2)
N(2)-C(1)	1.357(2)
N(2)-N(1)	1.364(2)
N(2)-C(9)	1.418(2)
N(1)-C(7)	1.340(3)
C(3)-C(2)	1.353(3)
C(3)-C(4)	1.415(3)
C(3)-H(3)	0.9300
C(2)-C(6)	1.413(3)
C(2)-H(2)	0.9300
C(6)-C(1)	1.377(3)
C(6)-C(7)	1.417(3)
C(1)-H(1)	0.9300
C(9)-C(10)	1.339(3)

C(9)-C(8)	1.459(3)
C(10)-C(11)	1.427(3)
C(10)-H(10)	0.9300
C(11)-C(16)	1.384(3)
C(11)-C(12)	1.397(3)
C(12)-C(13)	1.371(3)
C(12)-H(12)	0.9300
C(13)-C(14)	1.385(3)
C(13)-H(13)	0.9300
C(14)-C(15)	1.376(3)
C(14)-H(14)	0.9300
C(4)-C(5)	1.357(3)
C(4)-H(4)	0.9300
C(5)-C(7)	1.415(3)
C(5)-H(5)	0.9300
C(16)-C(15)	1.379(3)
C(15)-H(15)	0.9300
C(8)-O(1)-C(16)	123.01(15)
C(1)-N(2)-N(1)	113.03(16)
C(1)-N(2)-C(9)	129.48(16)
N(1)-N(2)-C(9)	117.49(15)
C(7)-N(1)-N(2)	103.43(15)
C(2)-C(3)-C(4)	121.1(2)
C(2)-C(3)-H(3)	119.4
C(4)-C(3)-H(3)	119.4
C(3)-C(2)-C(6)	118.4(2)
C(3)-C(2)-H(2)	120.8
C(6)-C(2)-H(2)	120.8
C(1)-C(6)-C(2)	135.1(2)
C(1)-C(6)-C(7)	104.36(18)
C(2)-C(6)-C(7)	120.51(18)
N(2)-C(1)-C(6)	106.82(17)
N(2)-C(1)-H(1)	126.6
C(6)-C(1)-H(1)	126.6
C(10)-C(9)-N(2)	121.59(17)
C(10)-C(9)-C(8)	120.83(18)
N(2)-C(9)-C(8)	117.55(17)
C(9)-C(10)-C(11)	121.00(18)
C(9)-C(10)-H(10)	119.5
C(11)-C(10)-H(10)	119.5
C(16)-C(11)-C(12)	118.05(19)
C(16)-C(11)-C(10)	118.39(18)
C(12)-C(11)-C(10)	123.57(18)
C(13)-C(12)-C(11)	120.3(2)
C(13)-C(12)-H(12)	119.8
C(11)-C(12)-H(12)	119.8
C(12)-C(13)-C(14)	120.0(2)
C(12)-C(13)-H(13)	120.0
C(14)-C(13)-H(13)	120.0
C(15)-C(14)-C(13)	121.2(2)
C(15)-C(14)-H(14)	119.4
C(13)-C(14)-H(14)	119.4
C(5)-C(4)-C(3)	122.1(2)
C(5)-C(4)-H(4)	118.9
C(3)-C(4)-H(4)	118.9
C(4)-C(5)-C(7)	118.1(2)
C(4)-C(5)-H(5)	121.0
C(7)-C(5)-H(5)	121.0

N(1)-C(7)-C(5)	127.92(19)
N(1)-C(7)-C(6)	112.36(17)
C(5)-C(7)-C(6)	119.7(2)
C(15)-C(16)-O(1)	117.35(18)
C(15)-C(16)-C(11)	122.47(19)
O(1)-C(16)-C(11)	120.17(17)
O(2)-C(8)-O(1)	116.76(17)
O(2)-C(8)-C(9)	126.64(19)
O(1)-C(8)-C(9)	116.59(17)
C(14)-C(15)-C(16)	118.0(2)
C(14)-C(15)-H(15)	121.0
C(16)-C(15)-H(15)	121.0

[a] Symmetry transformations used to generate equivalent atoms.

Table S9. Anisotropic displacement parameters (Å^2 × 10^3) for **6**. The anisotropic displacement factor exponent takes the form: $-2\pi^2[h^2 a^{*2}U^{11} + ... + 2 h k a^* b^* U^{12}]$.

	U^{11}	U^{22}	U^{33}	U^{23}	U^{13}	U^{12}
O(1)	35(1)	58(1)	57(1)	2(1)	−6(1)	−3(1)
O(2)	35(1)	70(1)	76(1)	−7(1)	−4(1)	7(1)
N(2)	34(1)	53(1)	45(1)	4(1)	−2(1)	−1(1)
N(1)	33(1)	64(1)	51(1)	−2(1)	−4(1)	−2(1)
C(3)	73(2)	55(1)	58(1)	−6(1)	1(1)	−2(1)
C(2)	55(1)	60(1)	59(1)	−2(1)	4(1)	3(1)
C(6)	47(1)	50(1)	46(1)	4(1)	0(1)	0(1)
C(1)	38(1)	59(1)	56(1)	0(1)	4(1)	4(1)
C(9)	33(1)	51(1)	42(1)	7(1)	−2(1)	−3(1)
C(10)	32(1)	56(1)	50(1)	3(1)	−1(1)	−2(1)
C(11)	34(1)	53(1)	47(1)	5(1)	3(1)	−4(1)
C(12)	39(1)	64(1)	60(1)	0(1)	8(1)	−3(1)
C(13)	54(1)	64(2)	60(1)	−8(1)	15(1)	−5(1)
C(14)	60(2)	65(2)	55(1)	−6(1)	3(1)	−16(1)
C(4)	59(2)	67(2)	60(1)	−4(1)	−10(1)	−8(1)
C(5)	43(1)	70(2)	62(1)	−4(1)	−6(1)	−4(1)
C(7)	43(1)	52(1)	44(1)	6(1)	−2(1)	−1(1)
C(16)	39(1)	49(1)	46(1)	6(1)	4(1)	−5(1)
C(8)	36(1)	51(1)	49(1)	7(1)	0(1)	−4(1)
C(15)	46(1)	63(2)	54(1)	5(1)	−4(1)	−9(1)

Table S10. Hydrogen coordinates (×10^4) and isotropic displacement parameters (Å^2 × 10^3) for **6**.

	**x**	**y**	**z**	**U(eq)**
H(3)	2006	13,592	8251	74
H(2)	570	12,758	7725	69
H(1)	−61	10,695	6681	61
H(10)	2217	7757	5701	55
H(12)	2554	5767	4865	65
H(13)	1969	4322	4003	71
H(14)	320	4540	3605	72
H(4)	3603	12,706	7974	75
H(5)	3807	11,023	7146	70
H(15)	−770	6189	4073	65

II. Copies of ¹H and ¹³C NMR spectra

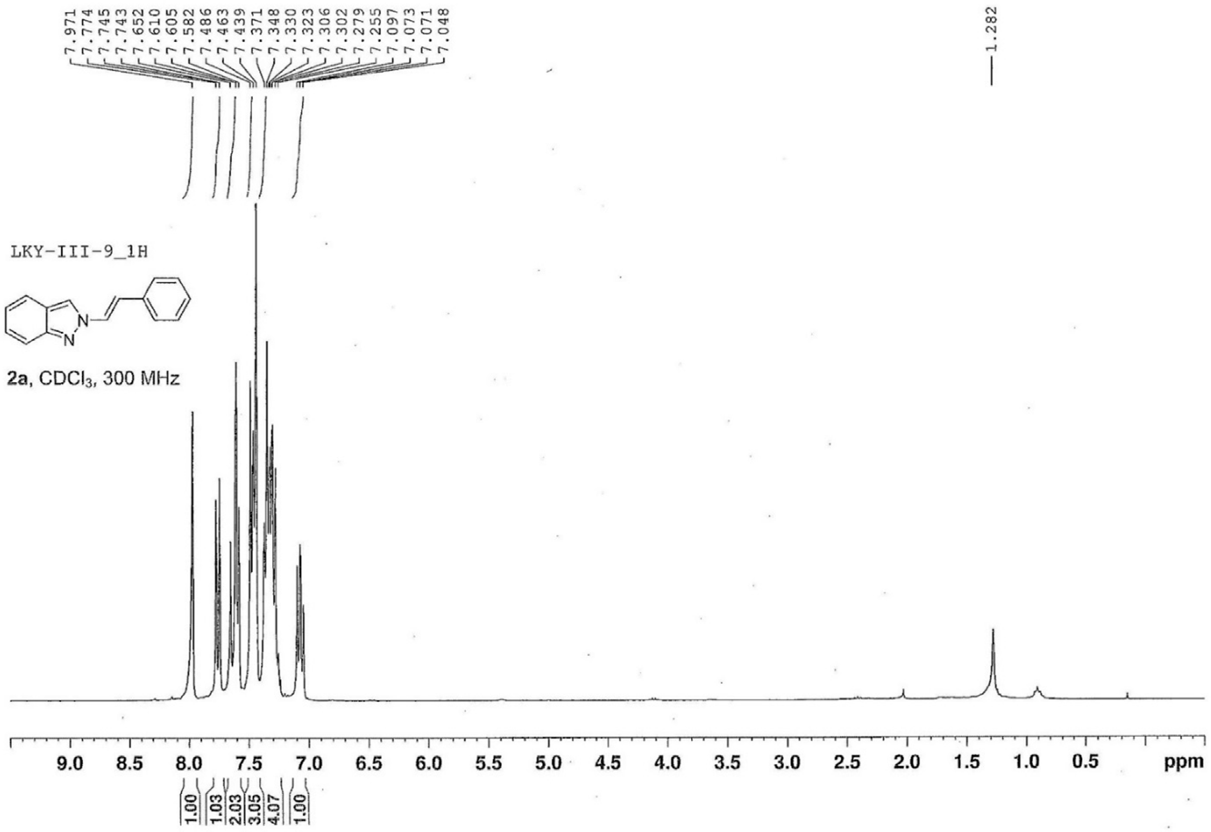

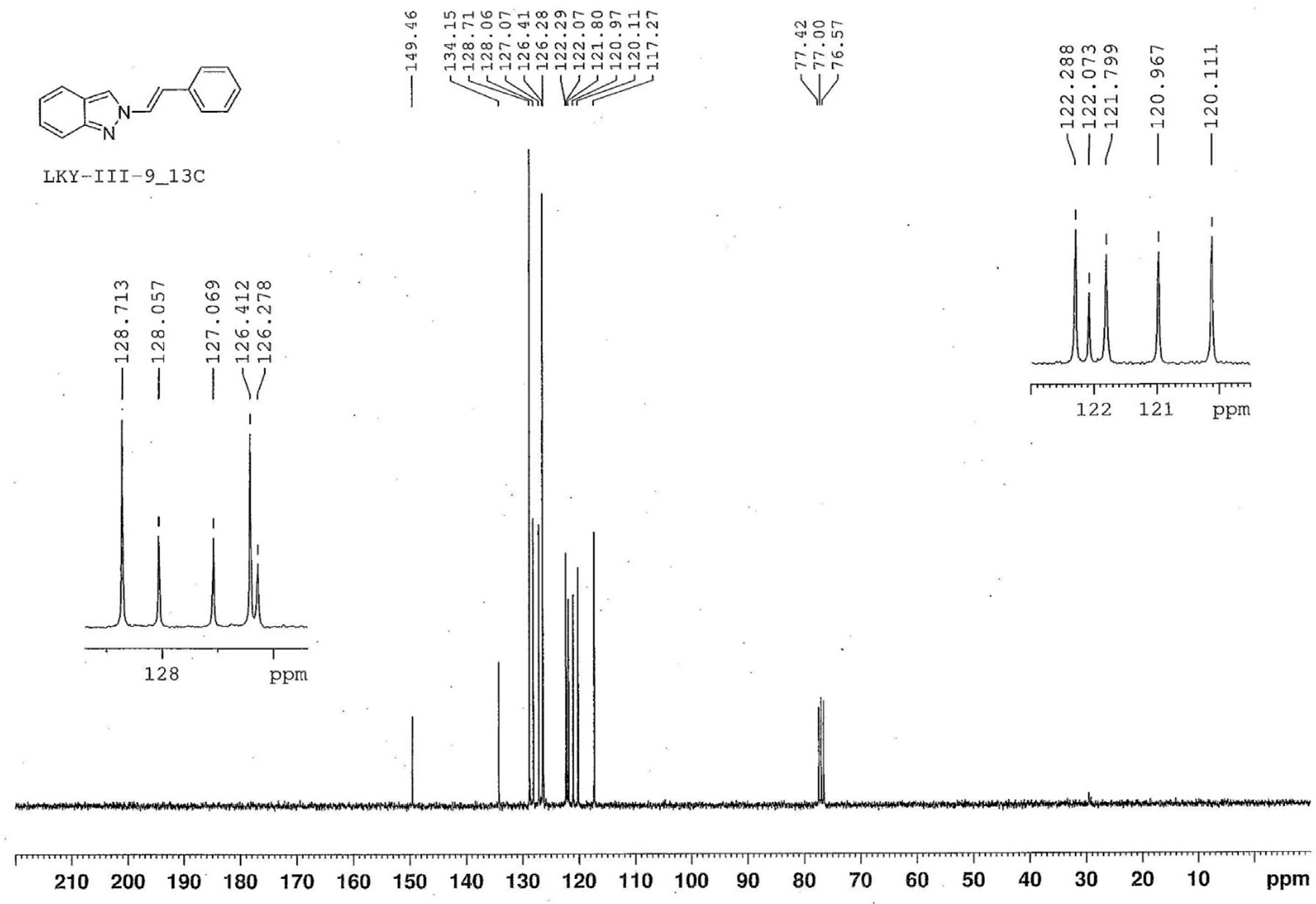
LKY-III-9_13C
149.46
134.15
128.71
128.06
127.07
126.41
126.28
122.29
122.07
121.80
120.97
120.11
117.27
77.42
77.00
76.57
128.713
128.057
127.069
126.412
126.278
128
ppm
122.288
122.073
121.799
120.967
120.111
122
121
ppm
210 200 190 180 170 160 150 140 130 120 110 100 90 80 70 60 50 40 30 20 10 ppm

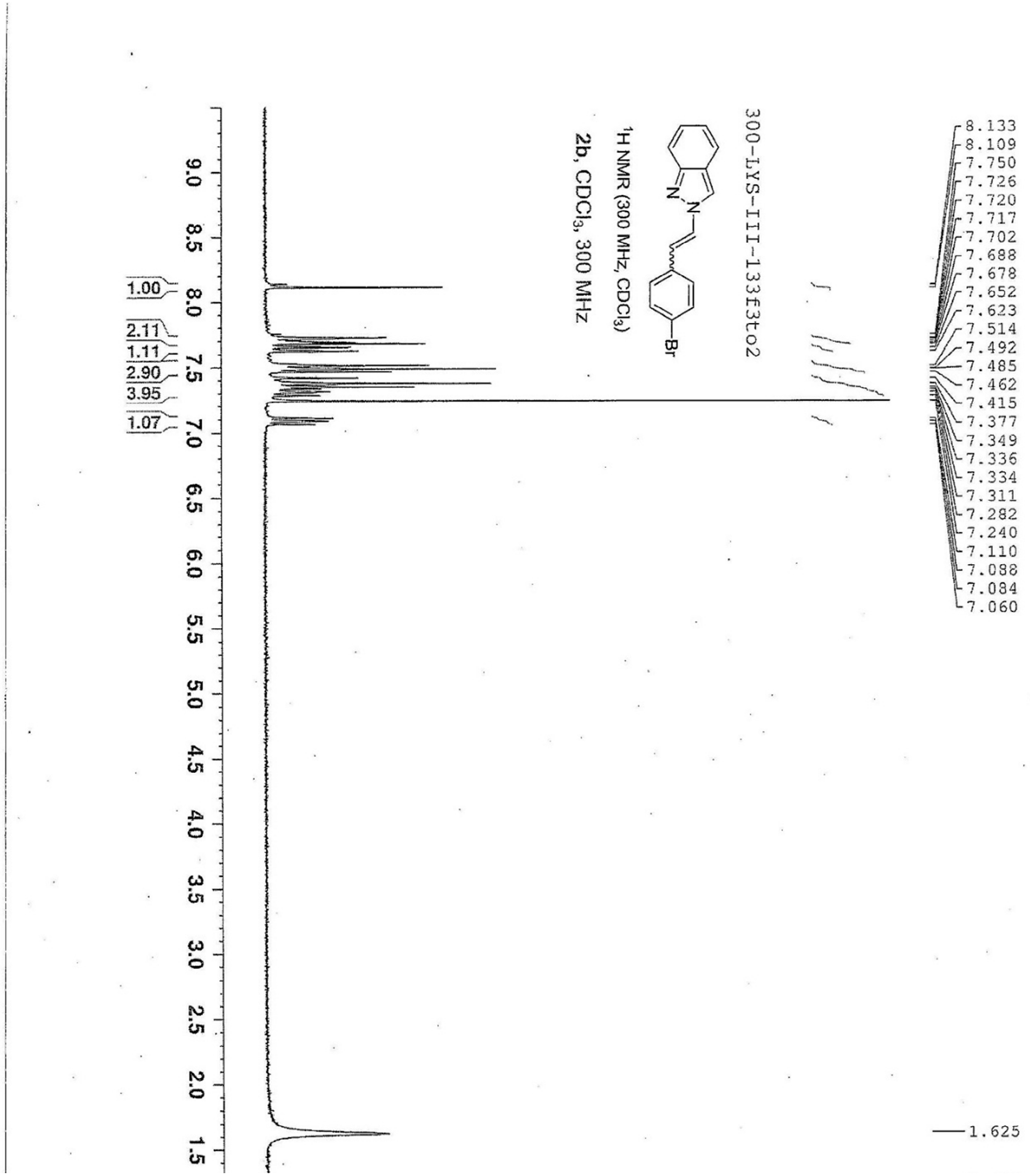
300-LYS-III-133f3to2
Br
N
N
¹H NMR (300 MHz, CDCl₃)
2b, CDCl₃, 300 MHz
8.133
8.109
7.750
7.726
7.720
7.717
7.702
7.688
7.678
7.652
7.623
7.514
7.492
7.485
7.462
7.415
7.377
7.349
7.336
7.334
7.311
7.282
7.240
7.110
7.088
7.084
7.060
1.625
1.00
2.11
1.11
2.90
3.95
1.07
9.0
8.5
8.0
7.5
7.0
6.5
6.0
5.5
5.0
4.5
4.0
3.5
3.0
2.5
2.0
1.5

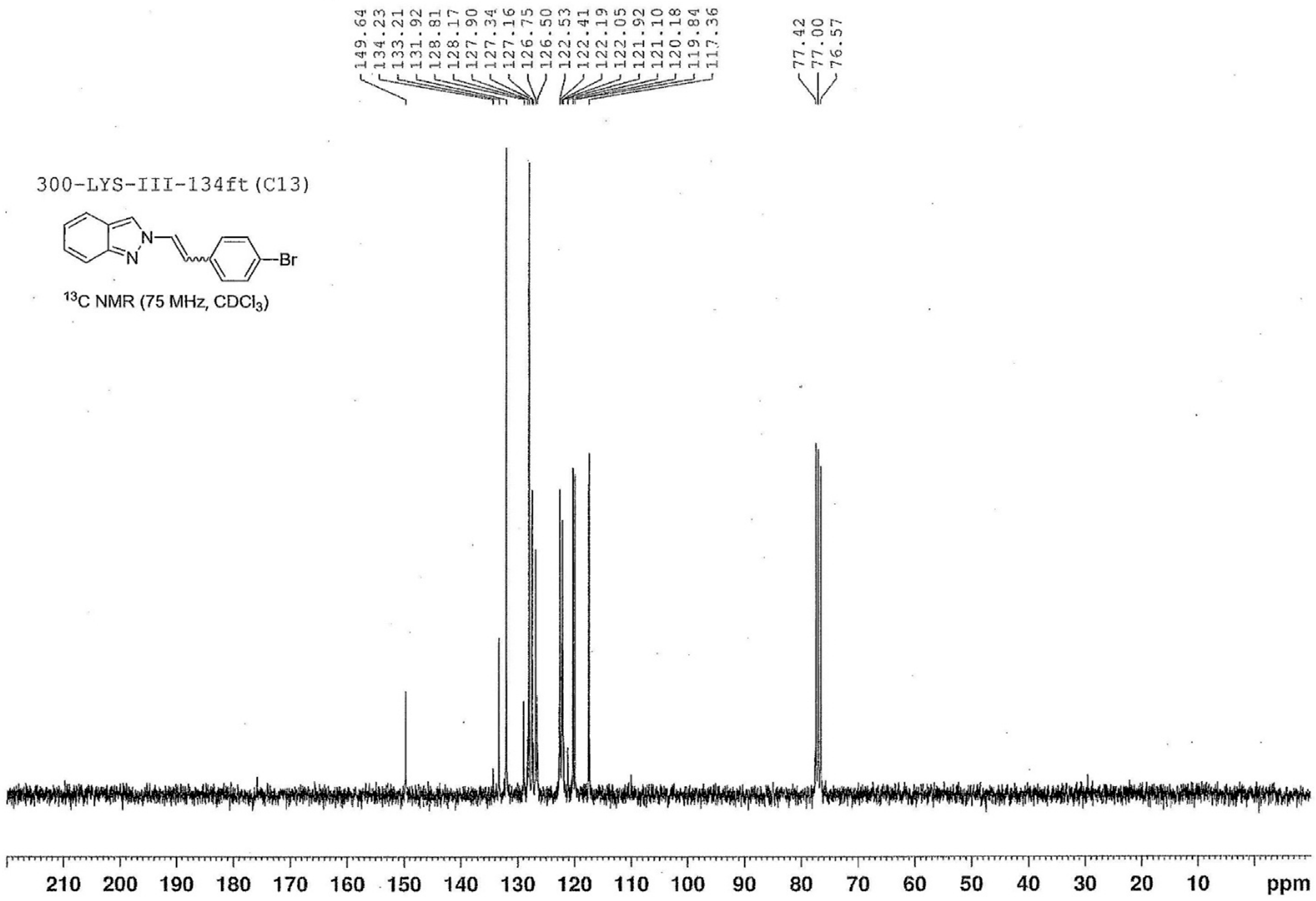
149.64
134.23
133.21
131.92
128.81
128.17
127.90
127.34
127.16
126.75
126.50
122.53
122.41
122.19
122.05
121.92
121.10
120.18
119.84
117.36
77.42
77.00
76.57
300-LYS-III-134ft(C13)
N
N
Br
^{13}C NMR (75 MHz, CDCl$_3$)
210 200 190 180 170 160 150 140 130 120 110 100 90 80 70 60 50 40 30 20 10 ppm

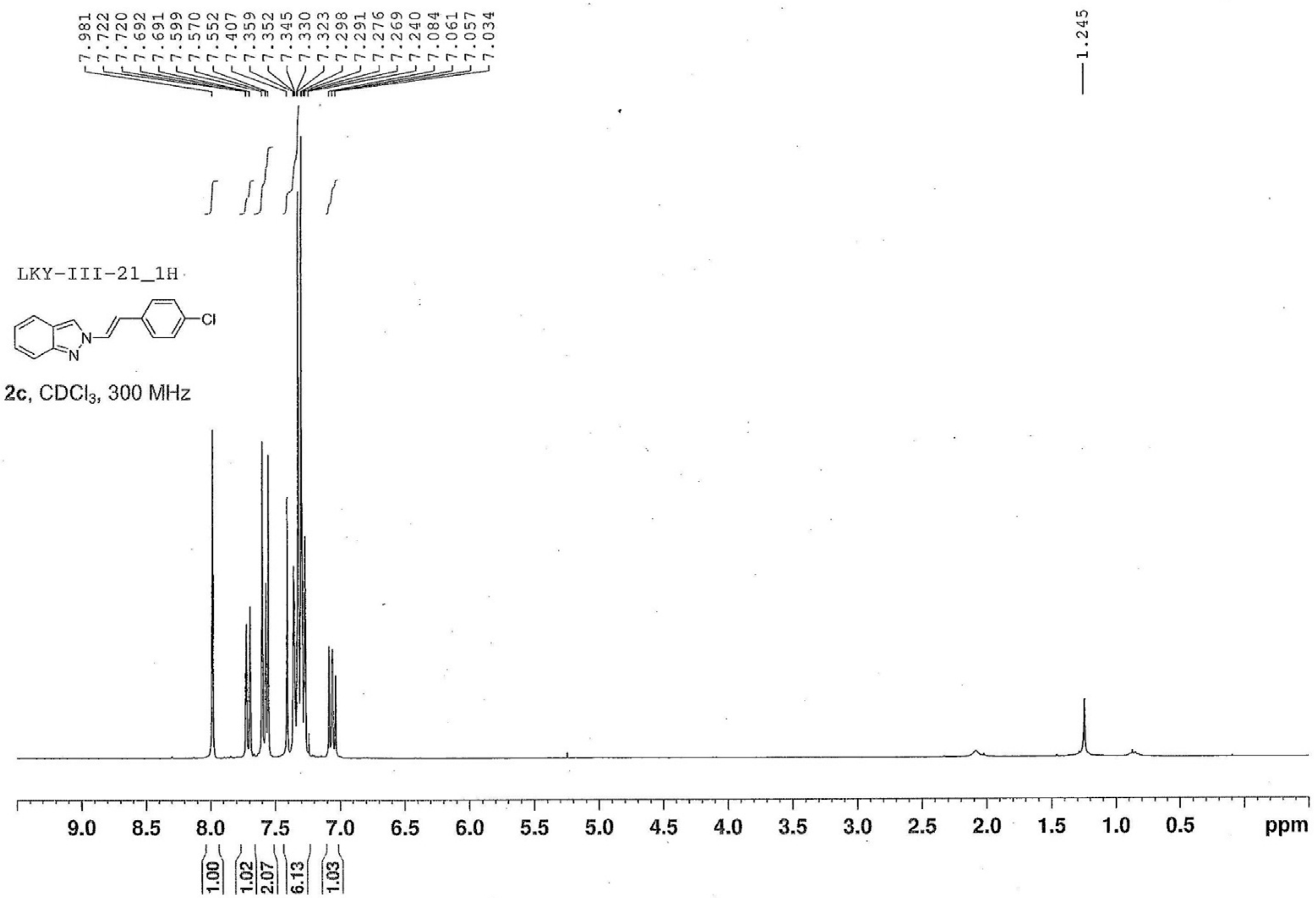

2c, $CDCl_3$, 300 MHz

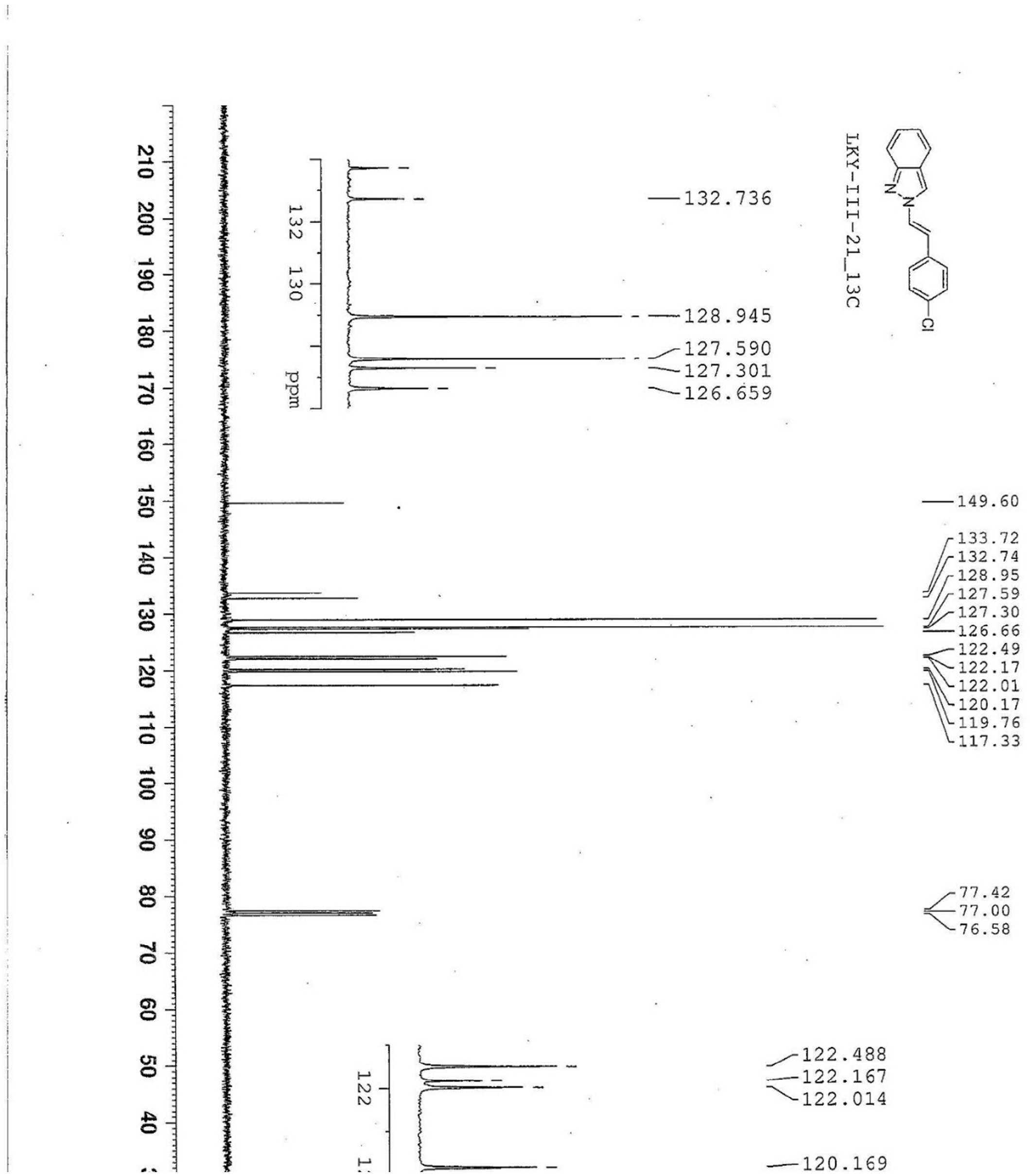
LKY-III-21_13C
Cl
N
132.736
128.945
127.590
127.301
126.659
132
130
ppm
149.60
133.72
132.74
128.95
127.59
127.30
126.66
122.49
122.17
122.01
120.17
119.76
117.33
77.42
77.00
76.58
122.488
122.167
122.014
120.169
122
210
200
190
180
170
160
150
140
130
120
110
100
90
80
70
60
50
40

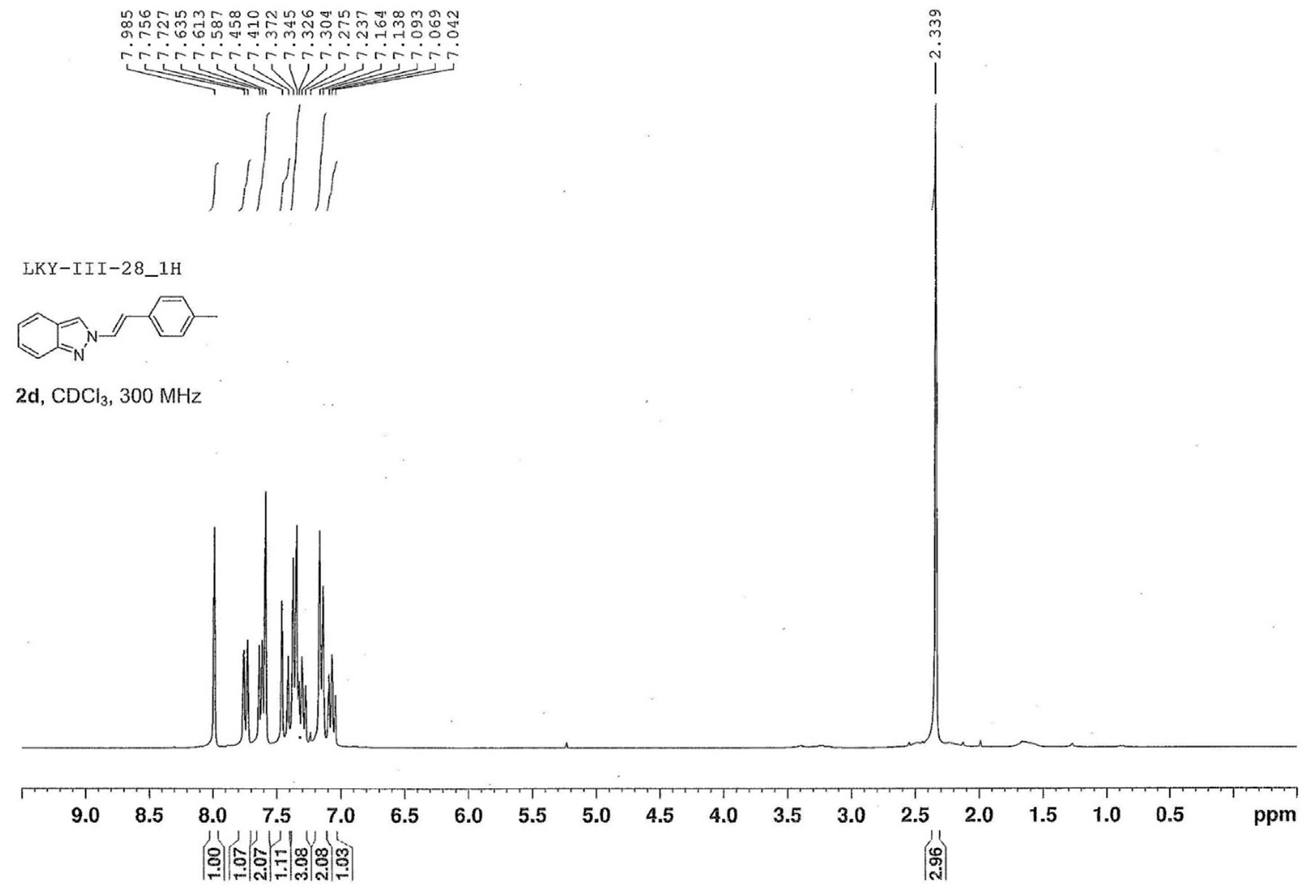
7.985
7.756
7.727
7.635
7.613
7.587
7.458
7.410
7.372
7.345
7.326
7.304
7.275
7.237
7.164
7.138
7.093
7.069
7.042
2.339
LKY-III-28_1H
N
N
2d, $CDCl_3$, 300 MHz
9.0
8.5
8.0
7.5
7.0
6.5
6.0
5.5
5.0
4.5
4.0
3.5
3.0
2.5
2.0
1.5
1.0
0.5
ppm
1.00
1.07
2.07
1.11
3.08
2.08
1.03
2.96

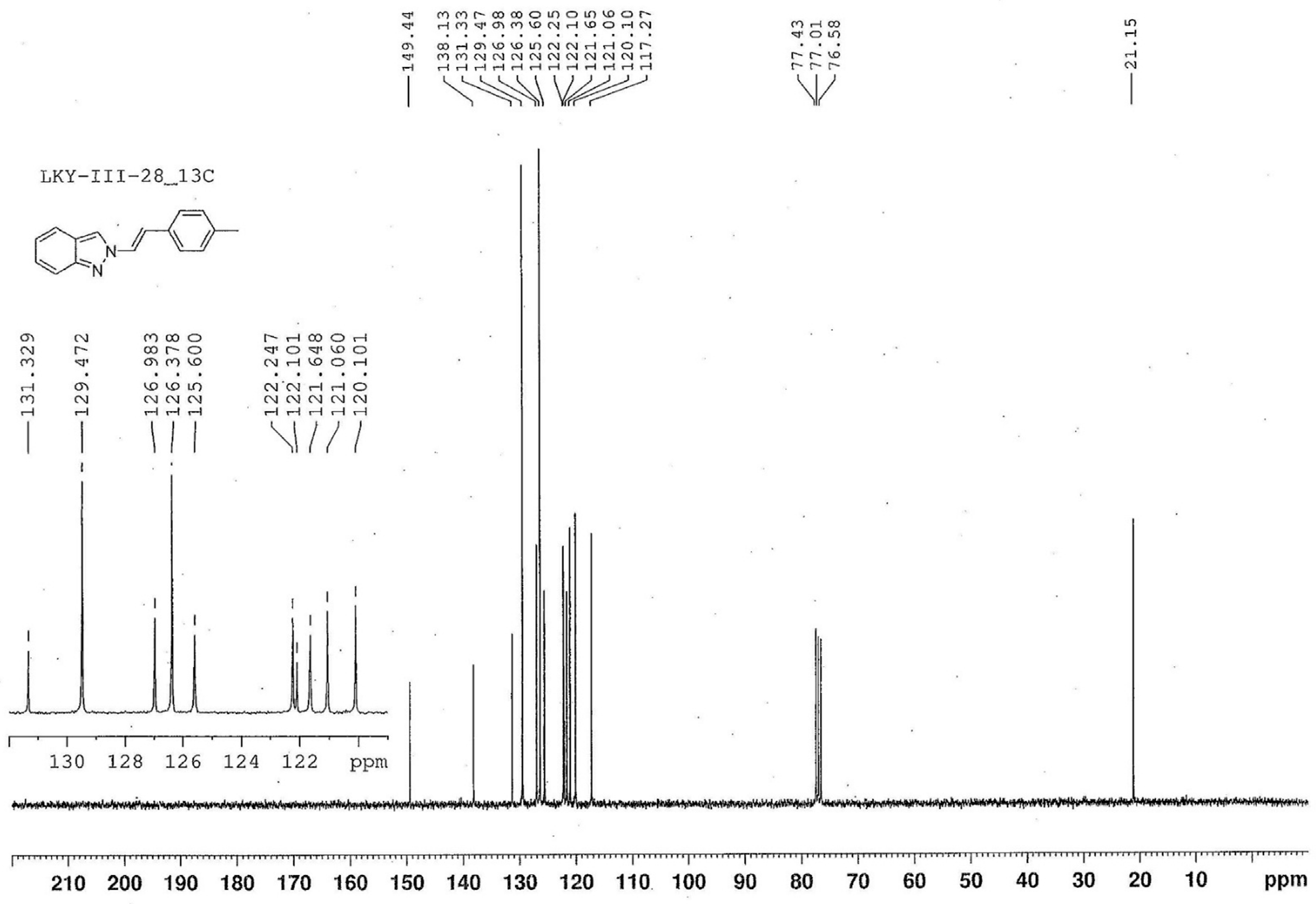

LKY-III-28_13C
149.44
138.13
131.33
129.47
126.98
126.38
125.60
122.25
122.10
121.65
121.06
120.10
117.27
77.43
77.01
76.58
21.15
131.329
129.472
126.983
126.378
125.600
122.247
122.101
121.648
121.060
120.101
130 128 126 124 122 ppm
210 200 190 180 170 160 150 140 130 120 110 100 90 80 70 60 50 40 30 20 10 ppm

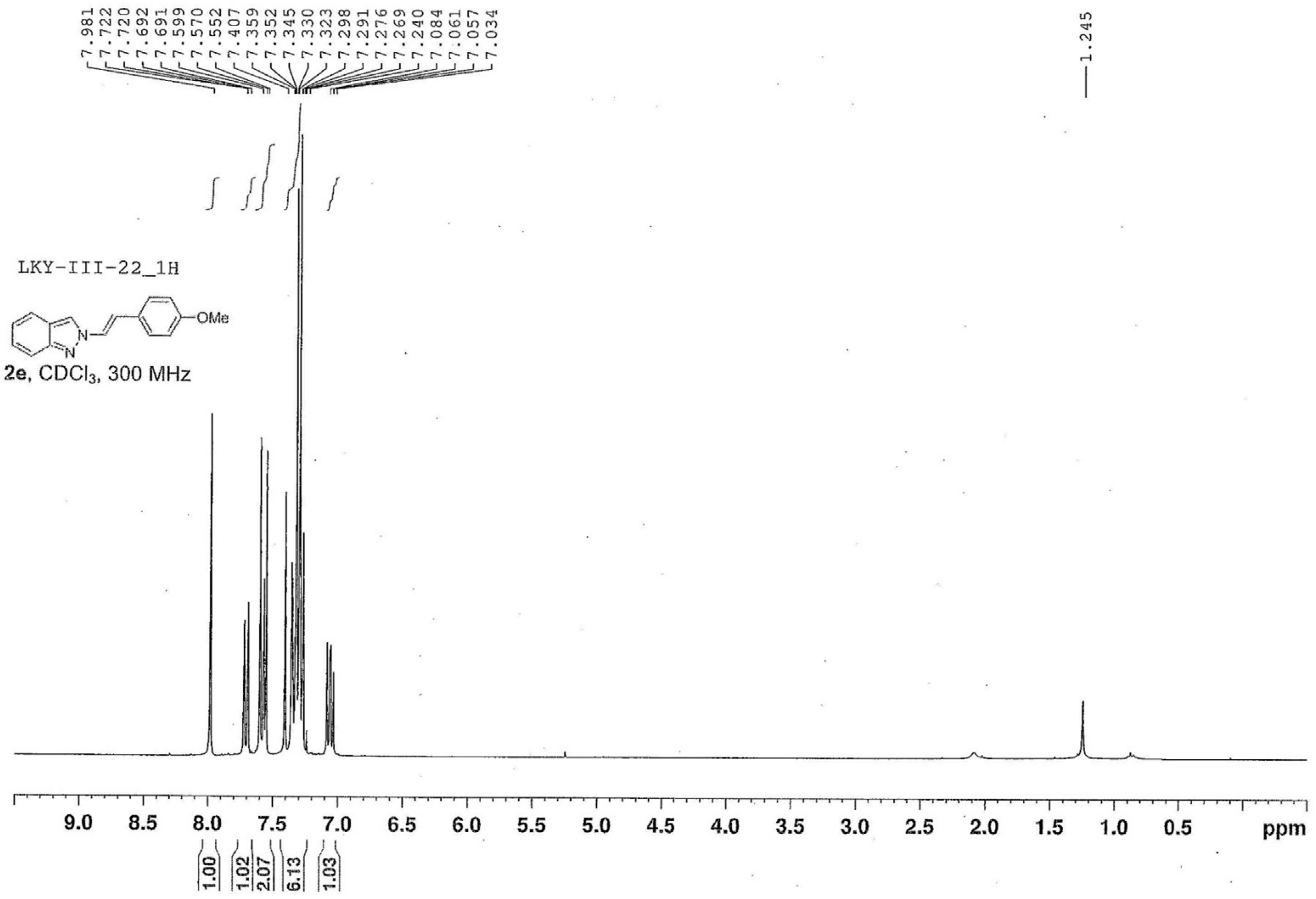
7.981
7.722
7.720
7.692
7.691
7.599
7.570
7.552
7.407
7.359
7.352
7.345
7.330
7.323
7.298
7.291
7.276
7.269
7.240
7.084
7.061
7.057
7.034
1.245
LKY-III-22_1H
OMe
N
N
2e, CDCl3, 300 MHz
9.0
8.5
8.0
7.5
7.0
6.5
6.0
5.5
5.0
4.5
4.0
3.5
3.0
2.5
2.0
1.5
1.0
0.5
ppm
1.00
1.02
2.07
6.13
1.03

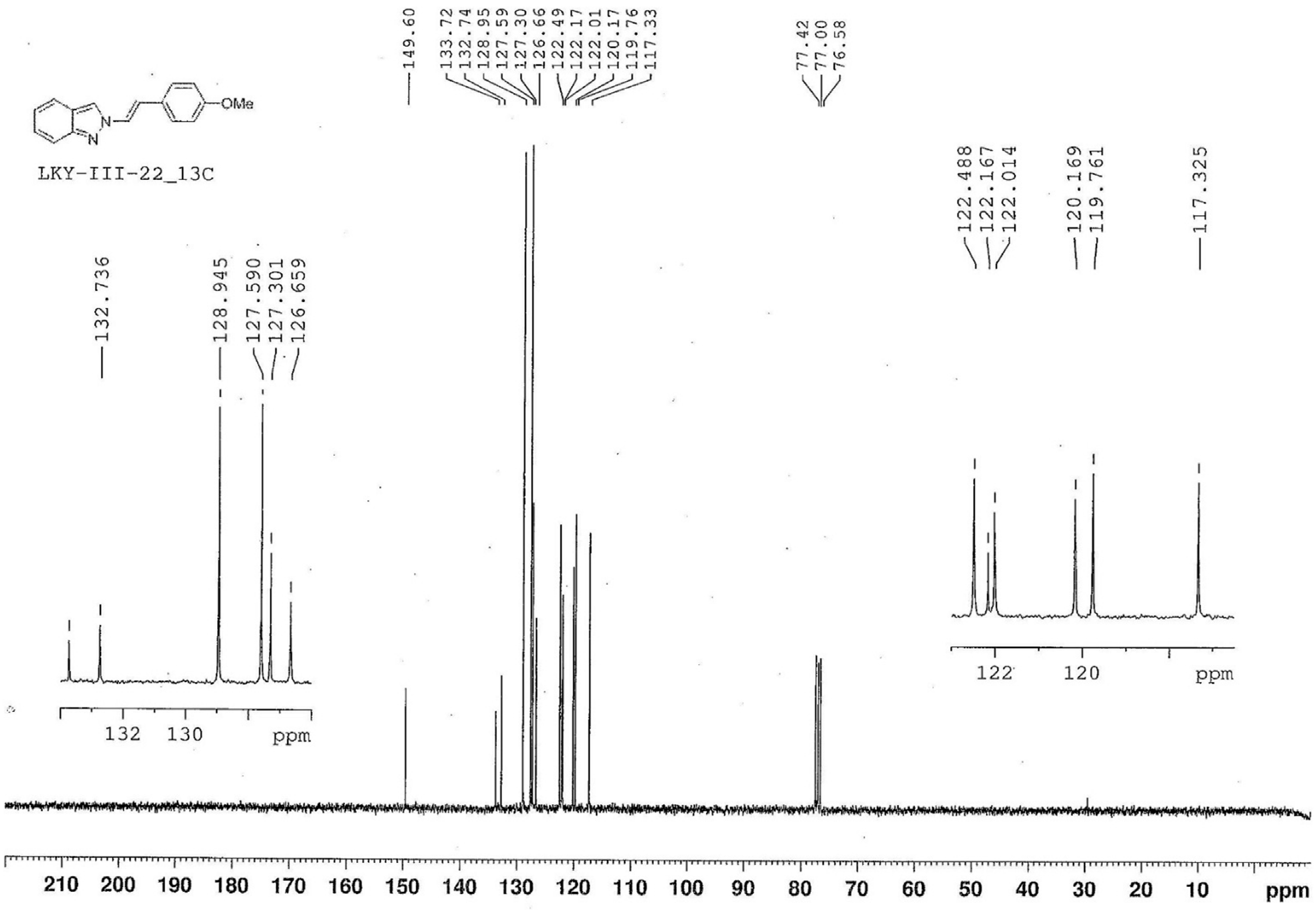

OMe
N
N
LKY-III-22_13C
149.60
133.72
132.74
128.95
127.59
127.30
126.66
122.49
122.17
122.01
120.17
119.76
117.33
77.42
77.00
76.58
132.736
128.945
127.590
127.301
126.659
132
130
ppm
122.488
122.167
122.014
120.169
119.761
117.325
122
120
ppm
210
200
190
180
170
160
150
140
130
120
110
100
90
80
70
60
50
40
30
20
10
ppm

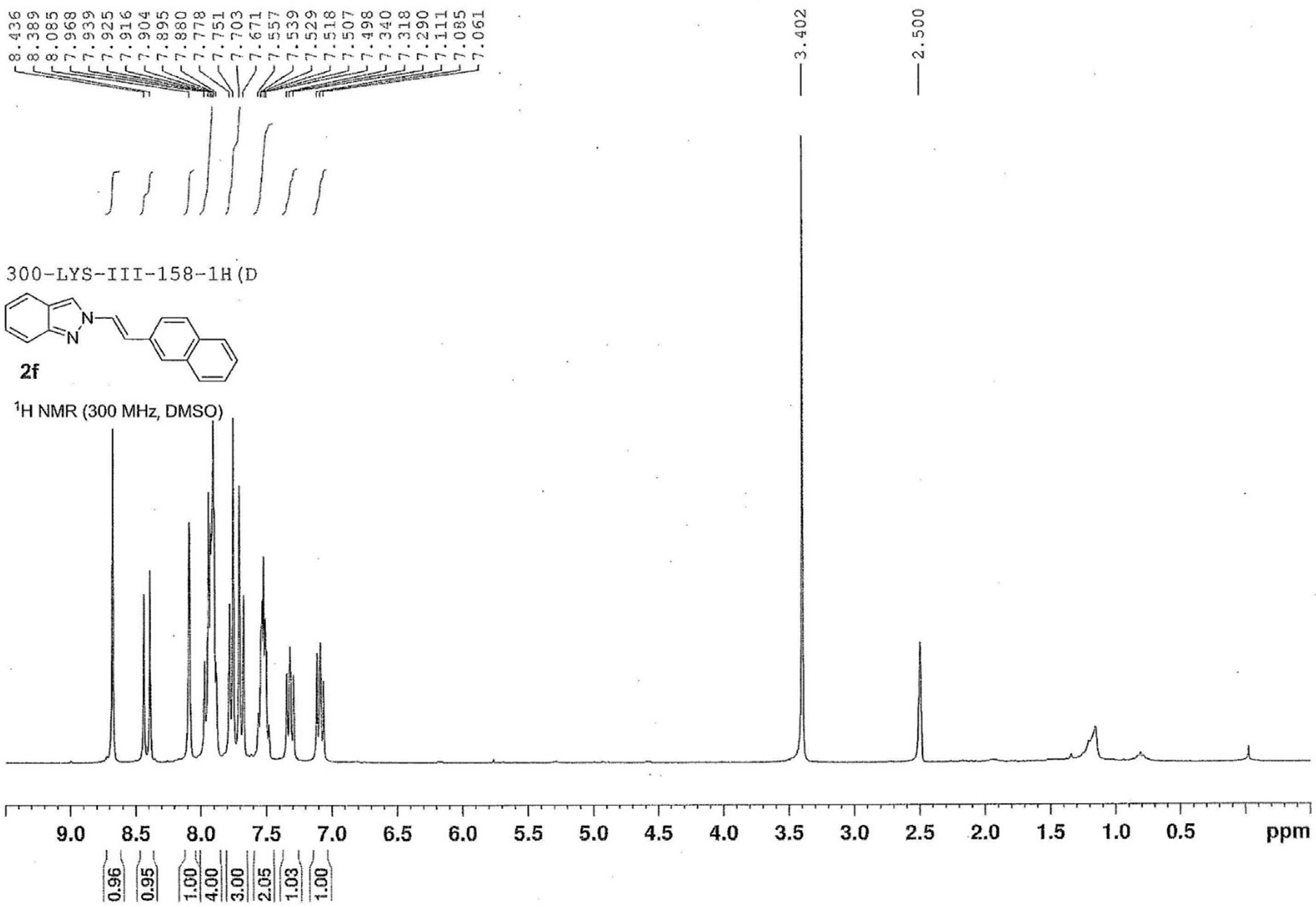
8.436
8.389
8.085
7.968
7.939
7.925
7.916
7.904
7.895
7.880
7.778
7.751
7.703
7.671
7.557
7.539
7.529
7.518
7.507
7.498
7.340
7.318
7.290
7.111
7.085
7.061
3.402
2.500
300-LYS-III-158-1H(D
2f
¹H NMR (300 MHz, DMSO)
9.0
8.5
8.0
7.5
7.0
6.5
6.0
5.5
5.0
4.5
4.0
3.5
3.0
2.5
2.0
1.5
1.0
0.5
ppm
0.96
0.95
1.00
4.00
3.00
2.05
1.03
1.00

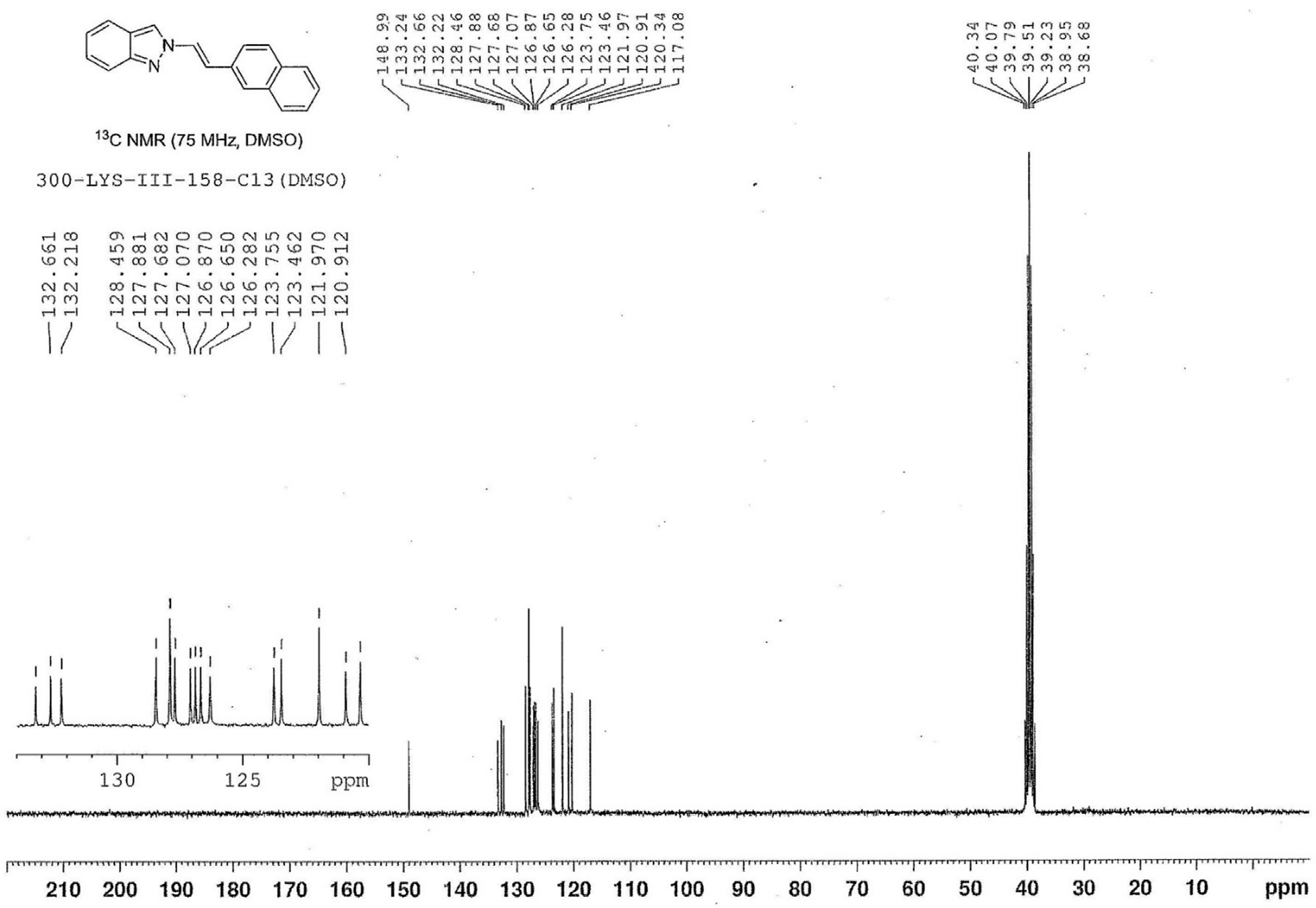
148.99
133.24
132.66
132.22
128.46
127.88
127.68
127.07
126.87
126.65
126.28
123.75
123.46
121.97
120.91
120.34
117.08
40.34
40.07
39.79
39.51
39.23
38.95
38.68
^{13}C NMR (75 MHz, DMSO)
300-LYS-III-158-C13(DMSO)
132.661
132.218
128.459
127.881
127.682
127.070
126.870
126.650
126.282
123.755
123.462
121.970
120.912
130
125
ppm
210 200 190 180 170 160 150 140 130 120 110 100 90 80 70 60 50 40 30 20 10 ppm

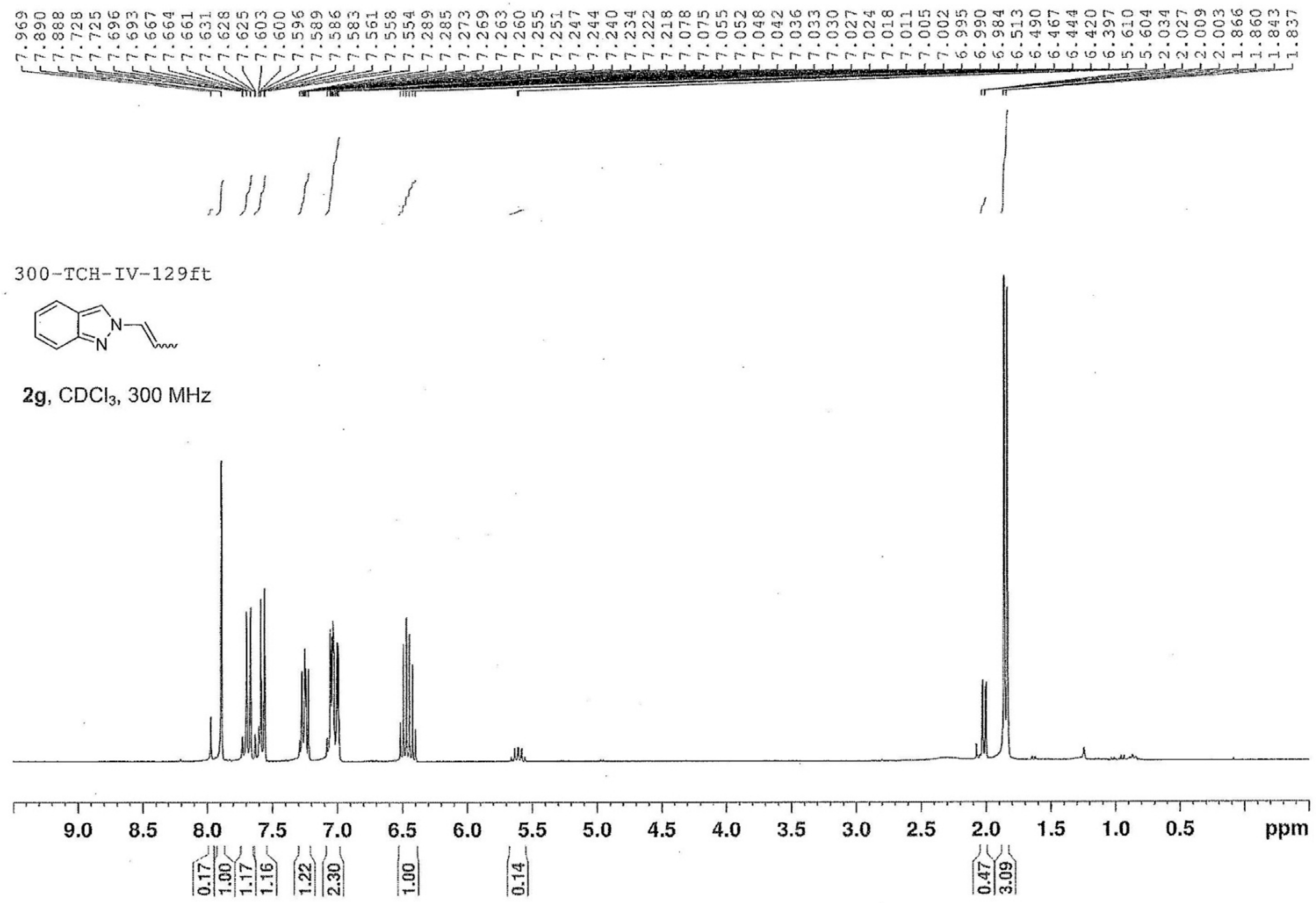
7.969 7.890 7.888 7.728 7.725 7.696 7.693 7.667 7.664 7.661 7.631 7.628 7.625 7.603 7.600 7.596 7.589 7.586 7.583 7.561 7.558 7.554 7.289 7.285 7.273 7.269 7.263 7.260 7.255 7.251 7.247 7.244 7.240 7.234 7.222 7.218 7.078 7.075 7.055 7.052 7.048 7.042 7.036 7.033 7.030 7.027 7.024 7.018 7.011 7.005 7.002 6.995 6.990 6.984 6.513 6.490 6.467 6.444 6.420 6.397 5.610 5.604 2.034 2.027 2.009 2.003 1.866 1.860 1.843 1.837
300-TCH-IV-129ft
N
N
2g, $CDCl_3$, 300 MHz
9.0 8.5 8.0 7.5 7.0 6.5 6.0 5.5 5.0 4.5 4.0 3.5 3.0 2.5 2.0 1.5 1.0 0.5 ppm
0.17 1.00 1.17 1.16 1.22 2.30 1.00 0.14 0.47 3.09

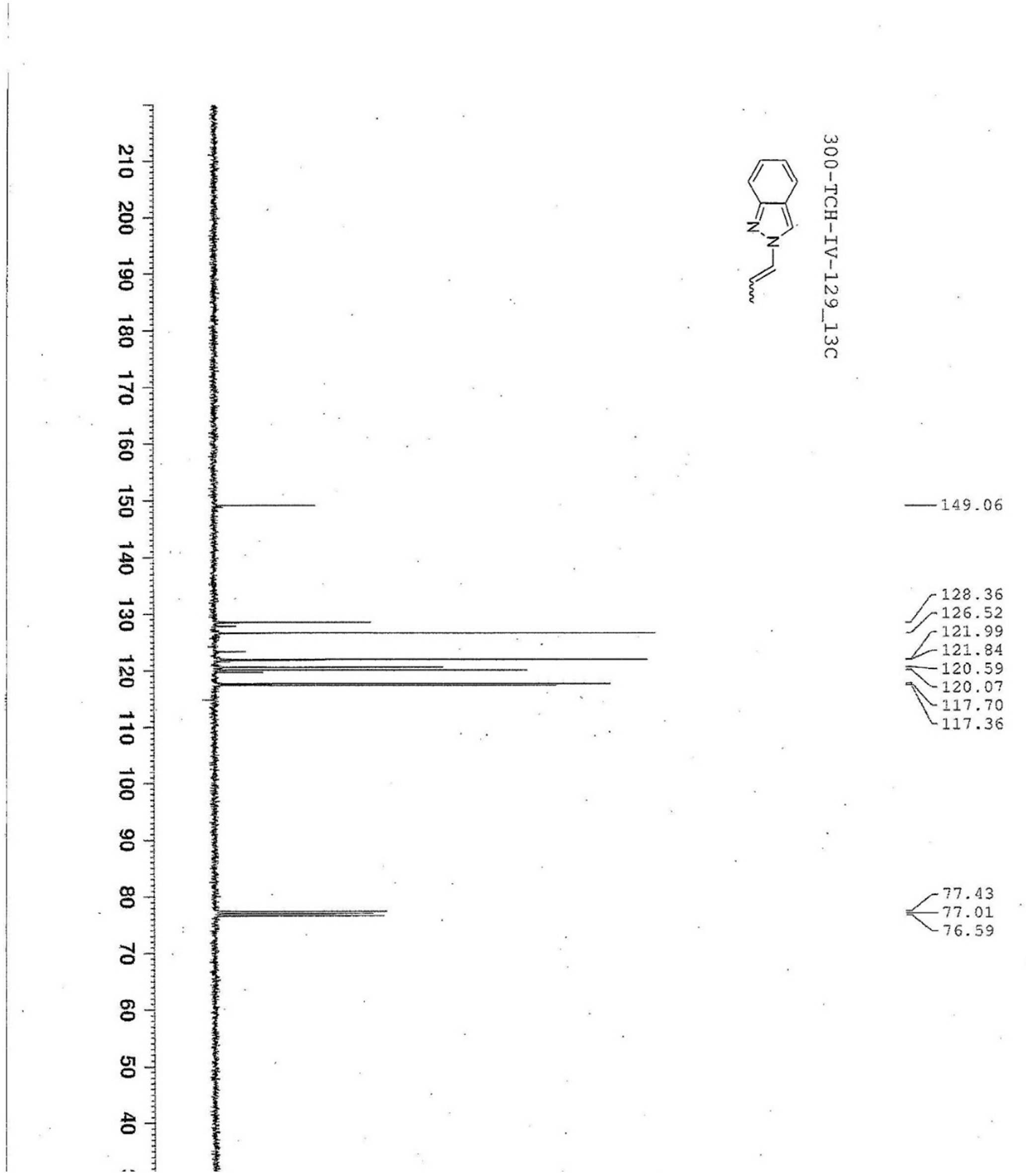
300-TCH-IV-129_13C
149.06
128.36
126.52
121.99
121.84
120.59
120.07
117.70
117.36
77.43
77.01
76.59
210
200
190
180
170
160
150
140
130
120
110
100
90
80
70
60
50
40

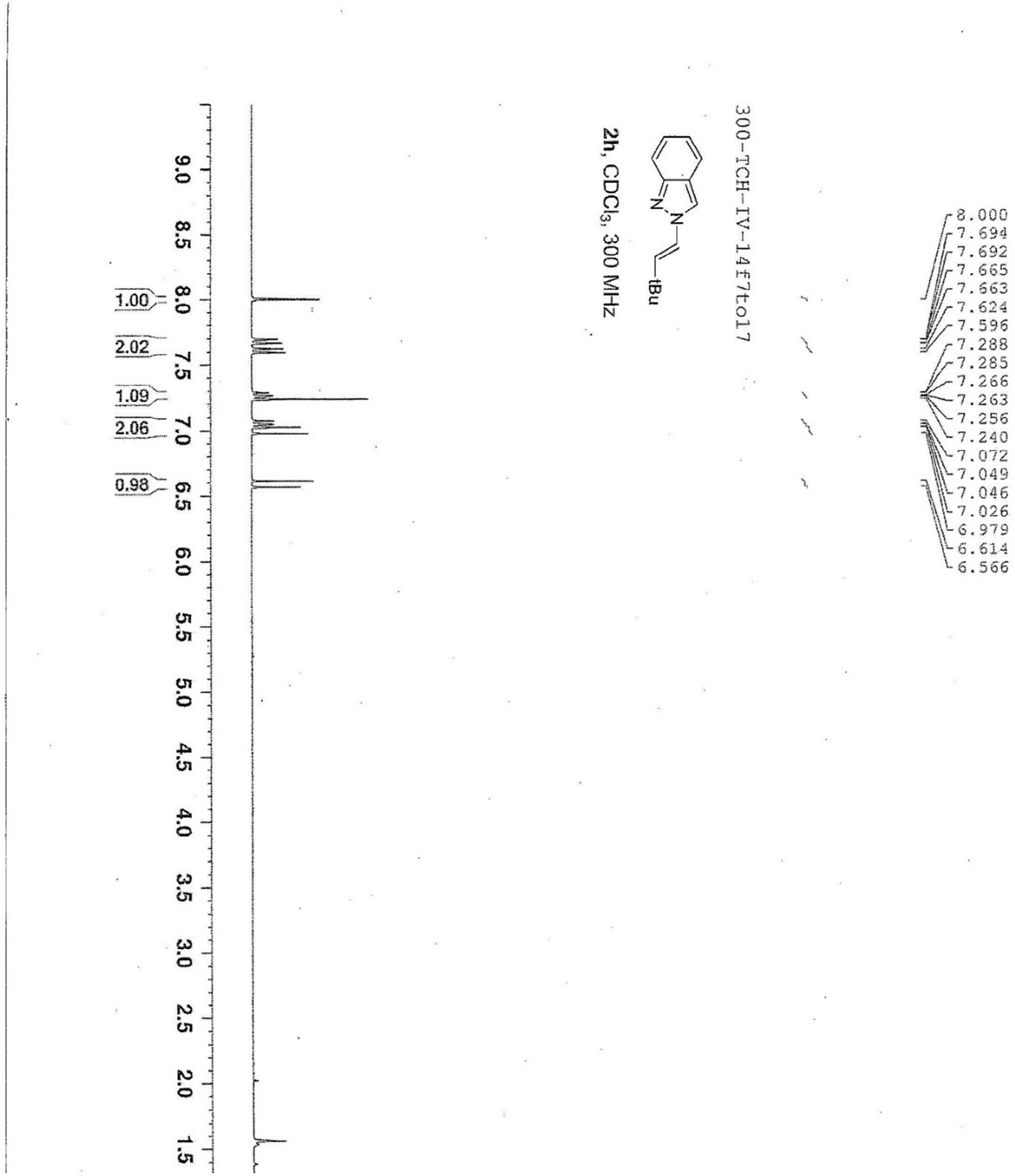
300-TCH-IV-14f7to17
2h, CDCl3, 300 MHz
tBu
8.000
7.694
7.692
7.665
7.663
7.624
7.596
7.288
7.285
7.266
7.263
7.256
7.240
7.072
7.049
7.046
7.026
6.979
6.614
6.566
1.00
2.02
1.09
2.06
0.98
9.0
8.5
8.0
7.5
7.0
6.5
6.0
5.5
5.0
4.5
4.0
3.5
3.0
2.5
2.0
1.5

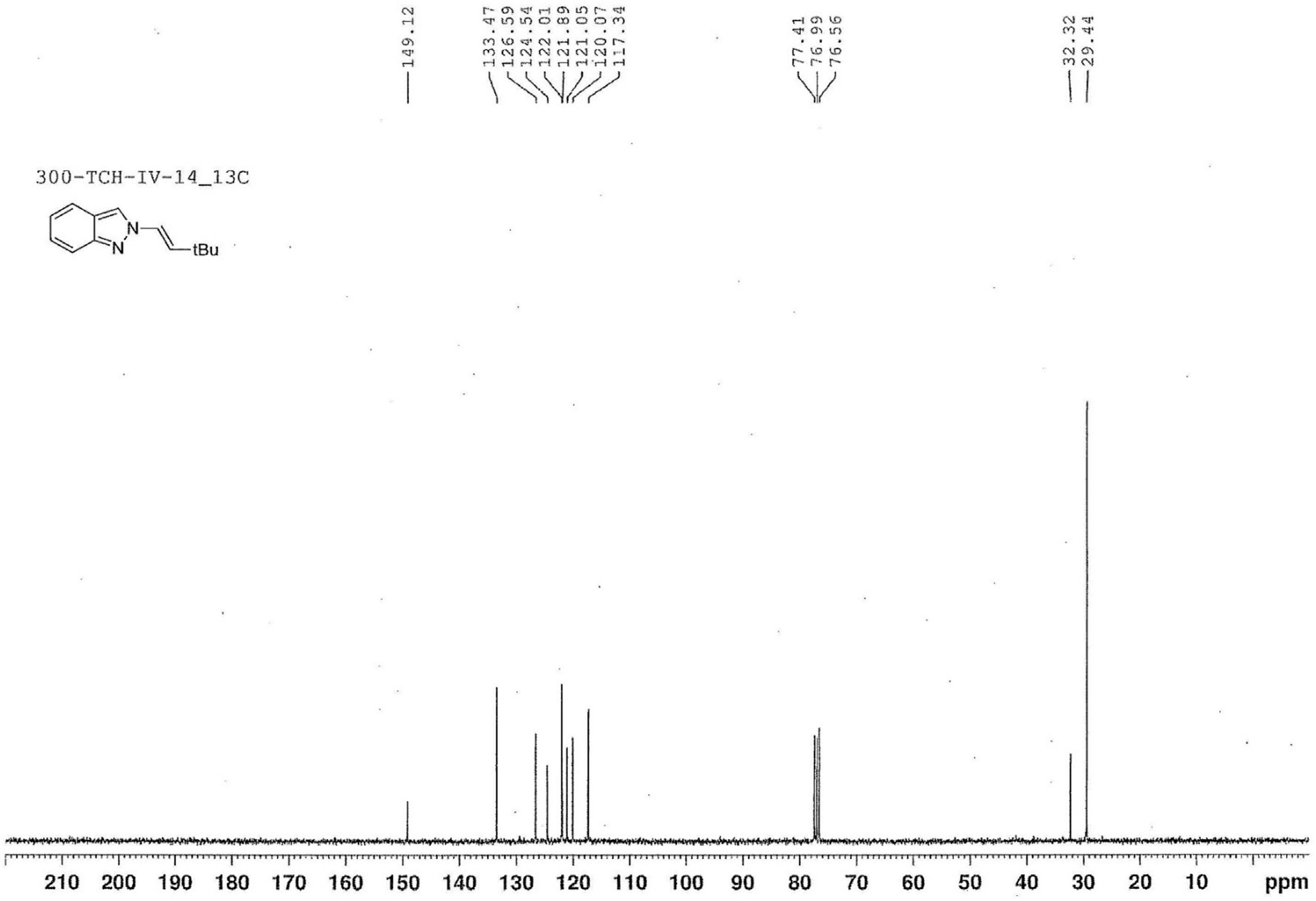
149.12
133.47
126.59
124.54
122.01
121.89
121.05
120.07
117.34
77.41
76.99
76.56
32.32
29.44
300-TCH-IV-14_13C
N
N
tBu
210
200
190
180
170
160
150
140
130
120
110
100
90
80
70
60
50
40
30
20
10
ppm

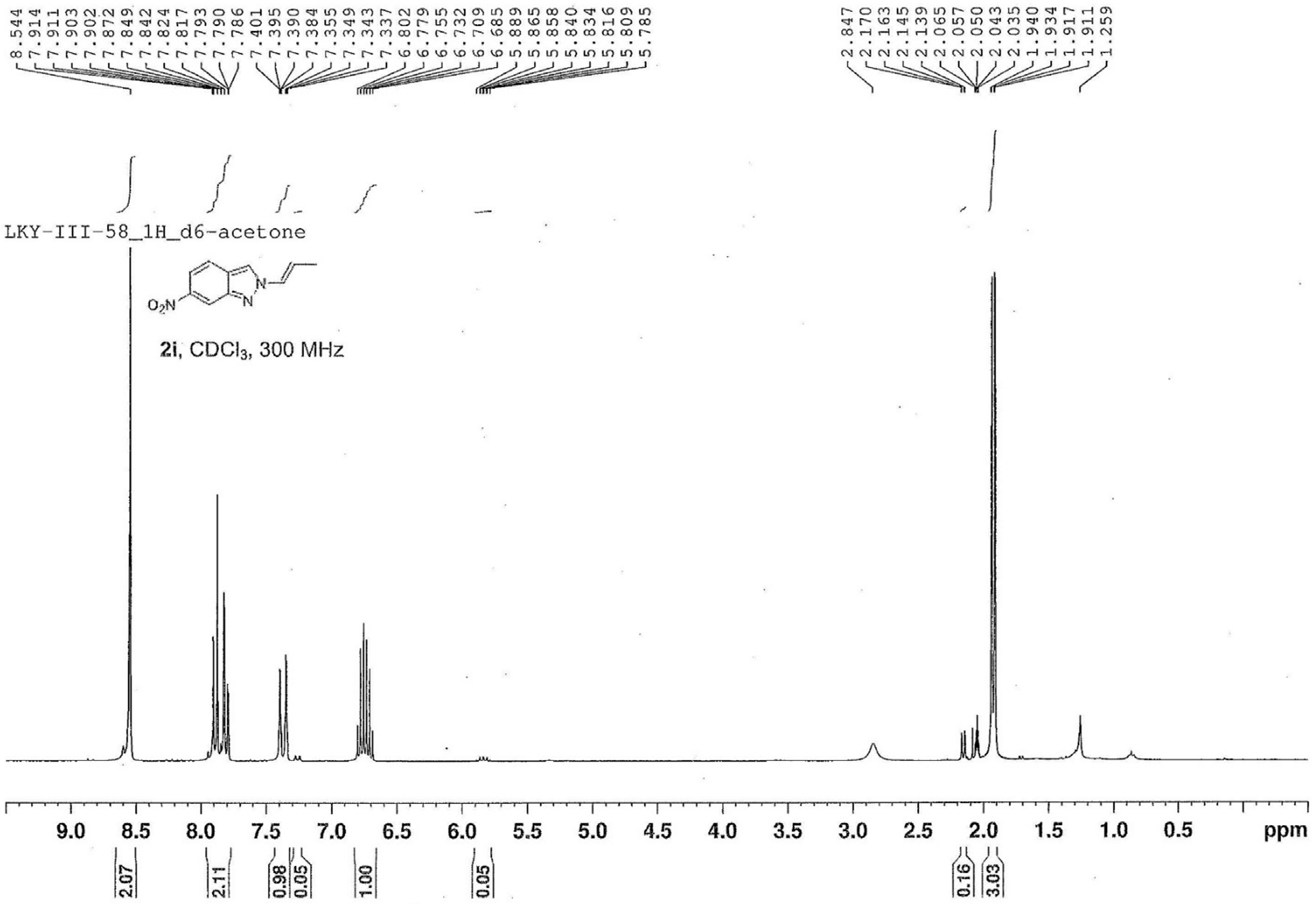
8.544
7.914
7.911
7.903
7.902
7.872
7.849
7.842
7.824
7.817
7.793
7.790
7.786
7.401
7.395
7.390
7.384
7.355
7.349
7.343
7.337
6.802
6.779
6.755
6.732
6.709
6.685
5.889
5.865
5.858
5.840
5.834
5.816
5.809
5.785
2.847
2.170
2.163
2.145
2.139
2.065
2.057
2.050
2.043
2.035
1.940
1.934
1.917
1.911
1.259
LKY-III-58_1H_d6-acetone
O_2N
N
N
2i, $CDCl_3$, 300 MHz
9.0
8.5
8.0
7.5
7.0
6.5
6.0
5.5
5.0
4.5
4.0
3.5
3.0
2.5
2.0
1.5
1.0
0.5
ppm
2.07
2.11
0.98
0.05
1.00
0.05
0.16
3.03

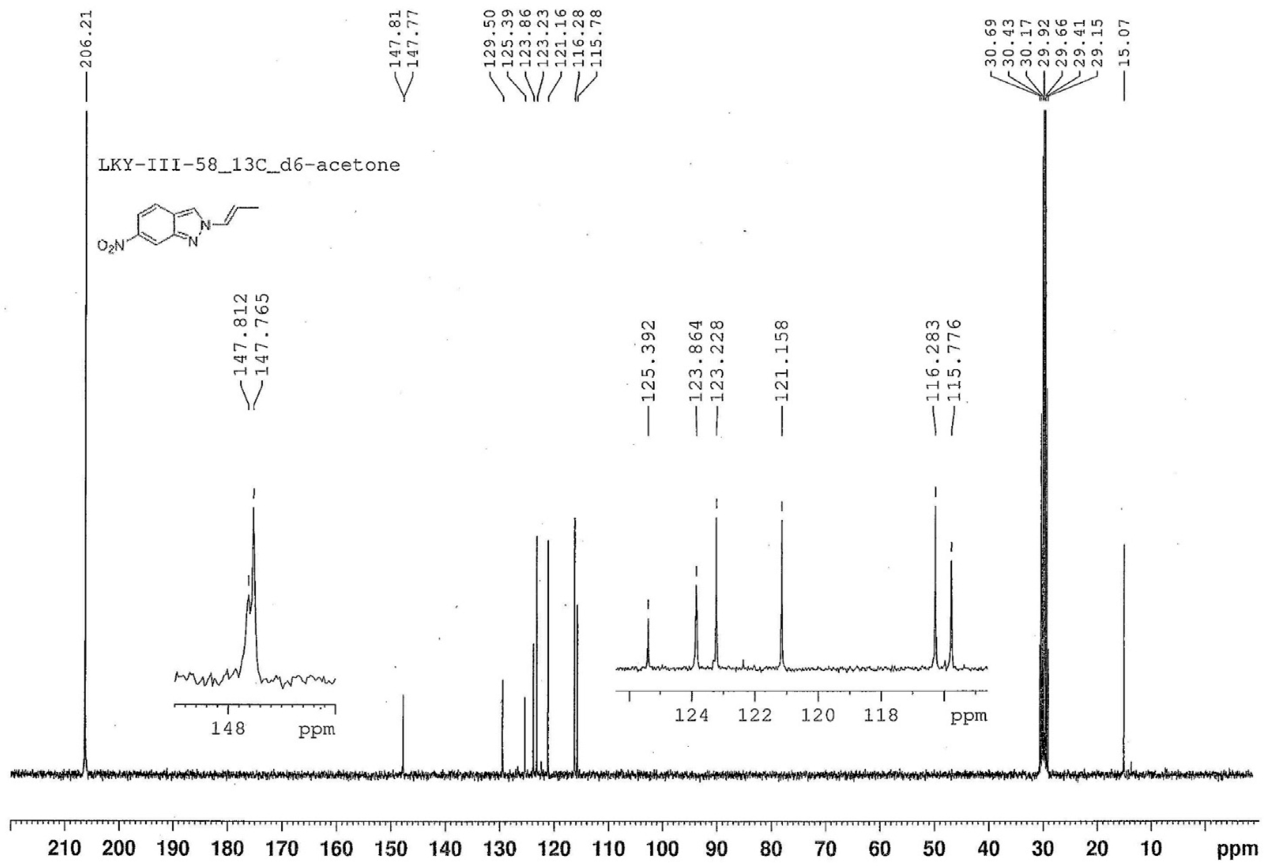

206.21
147.81
147.77
129.50
125.39
123.86
123.23
121.16
116.28
115.78
30.69
30.43
30.17
29.92
29.66
29.41
29.15
15.07
LKY-III-58_13C_d6-acetone
O_2N
N
147.812
147.765
148
ppm
125.392
123.864
123.228
121.158
116.283
115.776
124
122
120
118
ppm
210
200
190
180
170
160
150
140
130
120
110
100
90
80
70
60
50
40
30
20
10
ppm

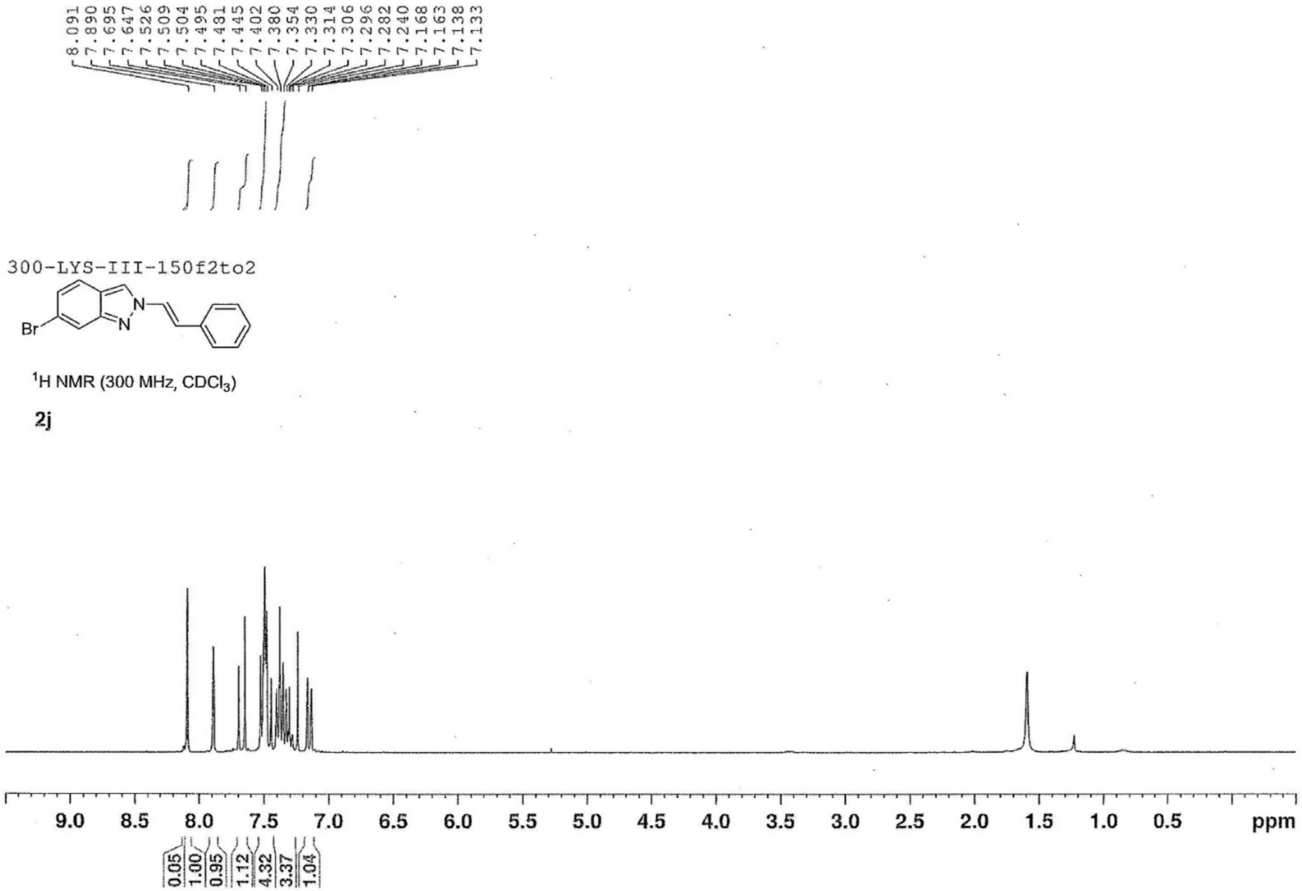
8.091
7.890
7.695
7.647
7.526
7.509
7.504
7.495
7.431
7.445
7.402
7.380
7.354
7.330
7.314
7.306
7.296
7.282
7.240
7.168
7.163
7.138
7.133
300-LYS-III-150f2to2
Br
N
N
1H NMR (300 MHz, CDCl3)
2j
9.0 8.5 8.0 7.5 7.0 6.5 6.0 5.5 5.0 4.5 4.0 3.5 3.0 2.5 2.0 1.5 1.0 0.5 ppm
0.05
1.00
0.95
1.12
4.32
3.37
1.04

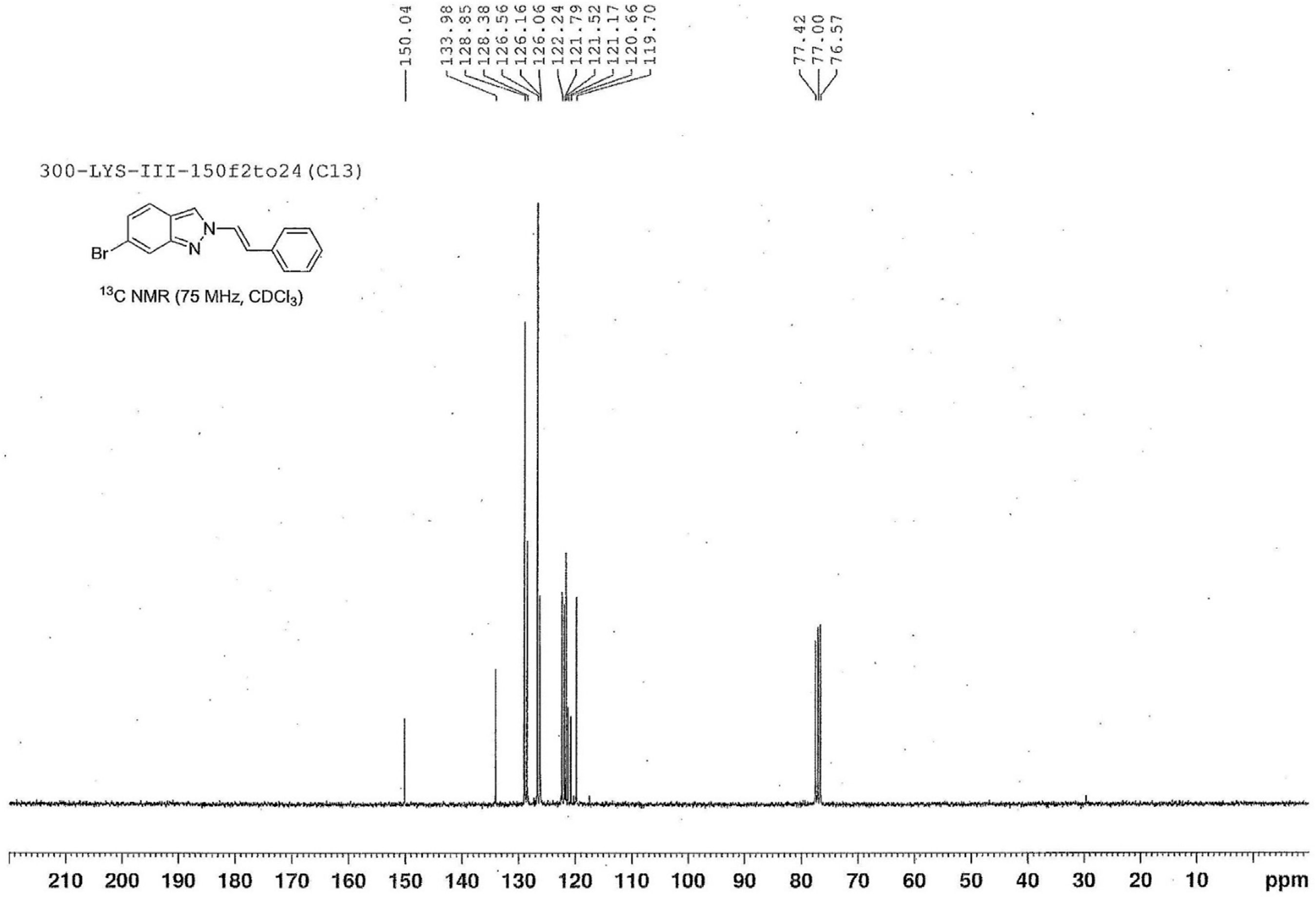
150.04
133.98
128.85
128.38
126.56
126.16
126.06
122.24
121.79
121.52
121.17
120.66
119.70
77.42
77.00
76.57
300-LYS-III-150f2to24(C13)
Br
N
N
13C NMR (75 MHz, CDCl3)
210 200 190 180 170 160 150 140 130 120 110 100 90 80 70 60 50 40 30 20 10 ppm

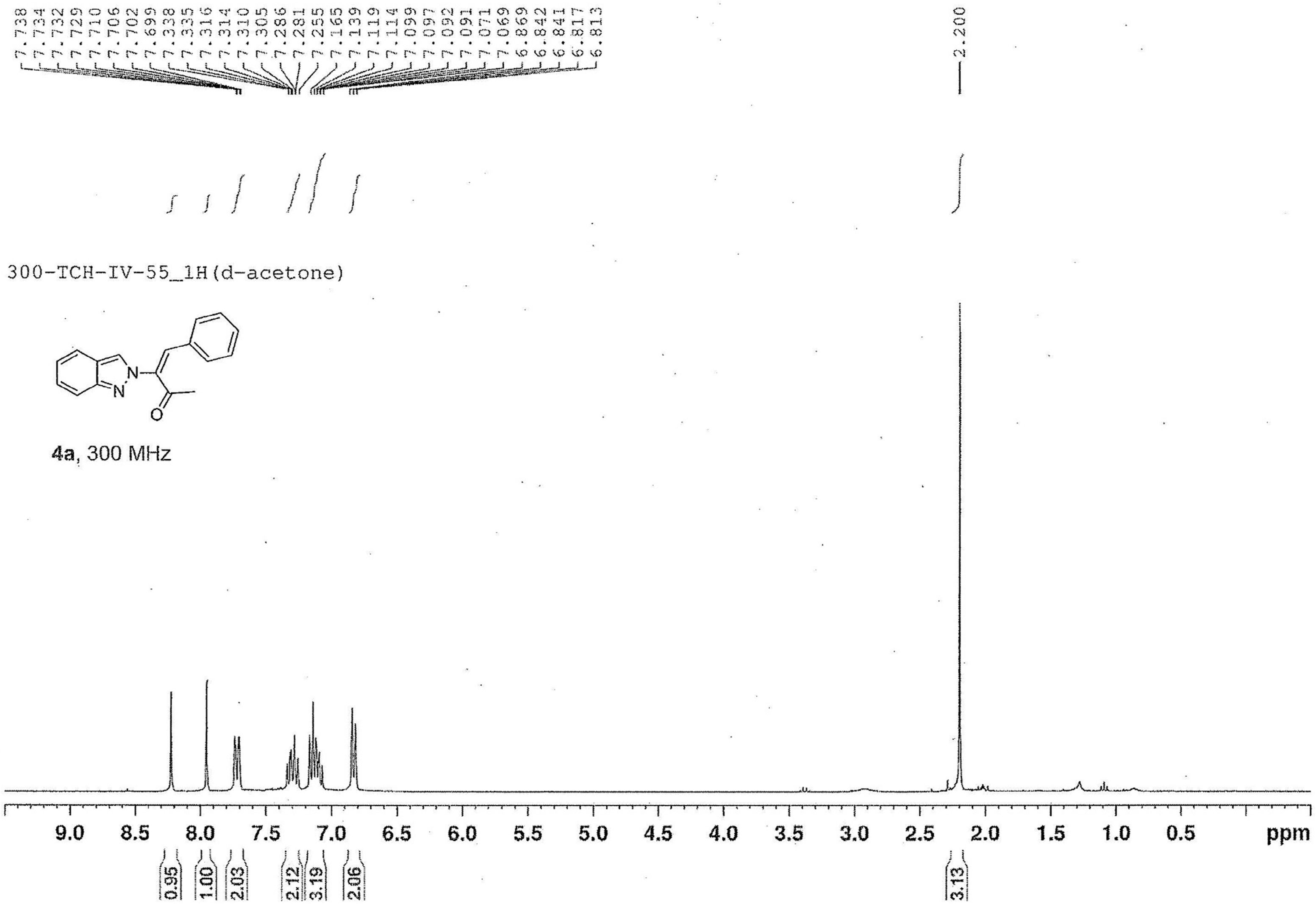
7.738
7.734
7.732
7.729
7.710
7.706
7.702
7.699
7.338
7.335
7.316
7.314
7.310
7.305
7.286
7.281
7.255
7.165
7.139
7.119
7.114
7.099
7.097
7.092
7.091
7.071
7.069
6.869
6.842
6.841
6.817
6.813
2.200
300-TCH-IV-55_1H(d-acetone)
N
N
O
9.0 8.5 8.0 7.5 7.0 6.5 6.0 5.5 5.0 4.5 4.0 3.5 3.0 2.5 2.0 1.5 1.0 0.5 ppm
0.95
1.00
2.03
2.12
3.19
2.06
3.13

4a, 300 MHz

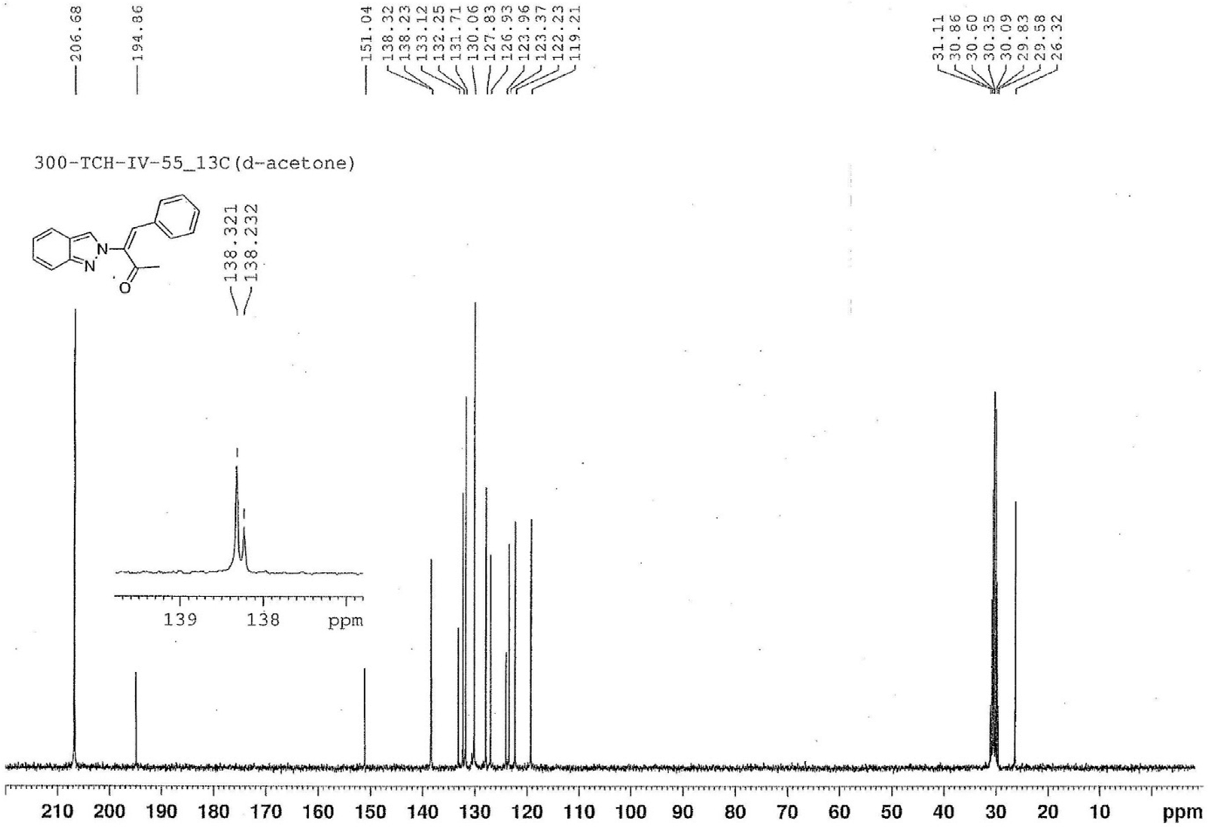
206.68
194.86
151.04
138.32
138.23
133.12
132.25
131.71
130.06
127.83
126.93
123.96
123.37
122.23
119.21
31.11
30.86
30.60
30.35
30.09
29.83
29.58
26.32
300-TCH-IV-55_13C(d-acetone)
138.321
138.232
139
138
ppm
210 200 190 180 170 160 150 140 130 120 110 100 90 80 70 60 50 40 30 20 10 ppm

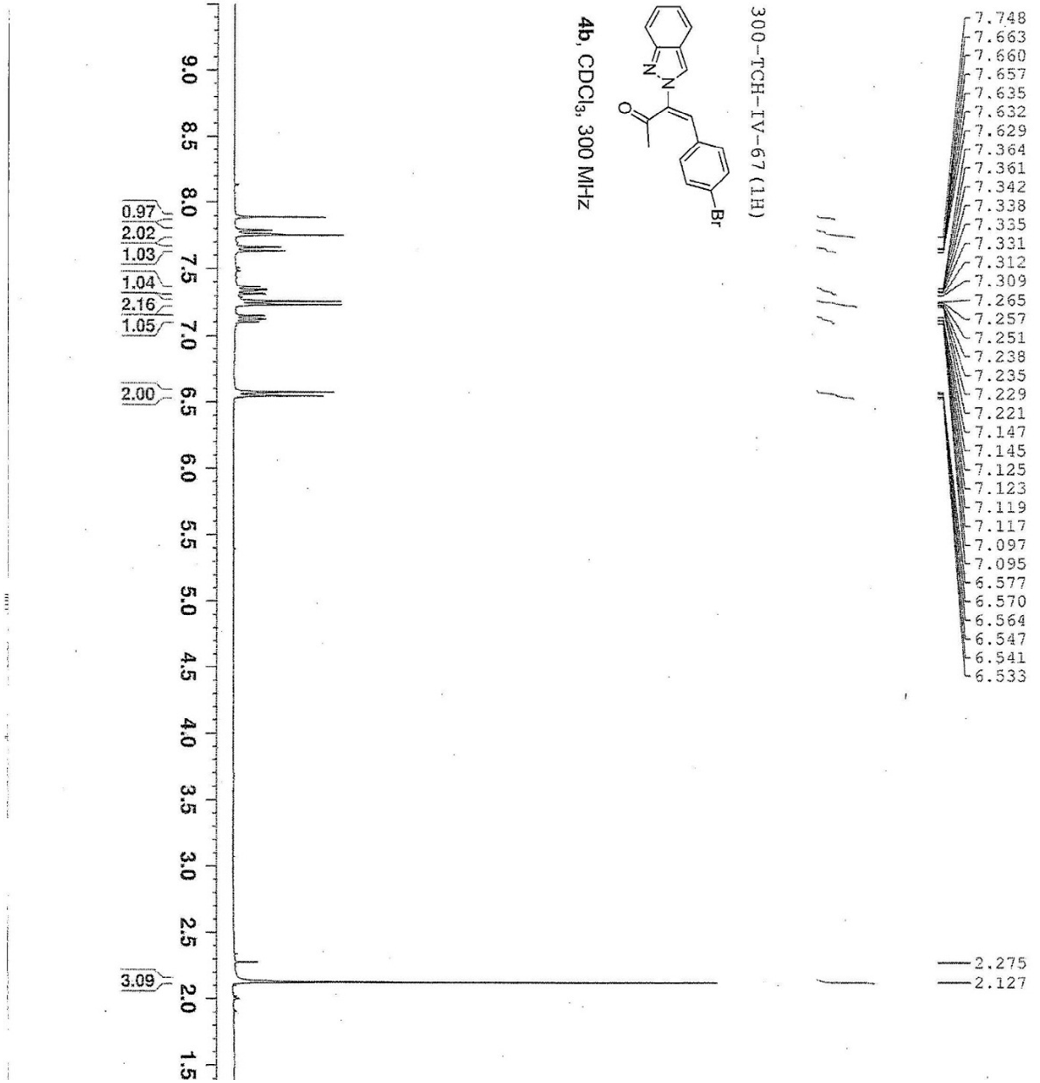
300-TCH-IV-67(1H)
4b, CDCl3, 300 MHz
Br
O
N
N
7.748
7.663
7.660
7.657
7.635
7.632
7.629
7.364
7.361
7.342
7.338
7.335
7.331
7.312
7.309
7.265
7.257
7.251
7.238
7.235
7.229
7.221
7.147
7.145
7.125
7.123
7.119
7.117
7.097
7.095
6.577
6.570
6.564
6.547
6.541
6.533
2.275
2.127
0.97
2.02
1.03
1.04
2.16
1.05
2.00
3.09
9.0
8.5
8.0
7.5
7.0
6.5
6.0
5.5
5.0
4.5
4.0
3.5
3.0
2.5
2.0
1.5

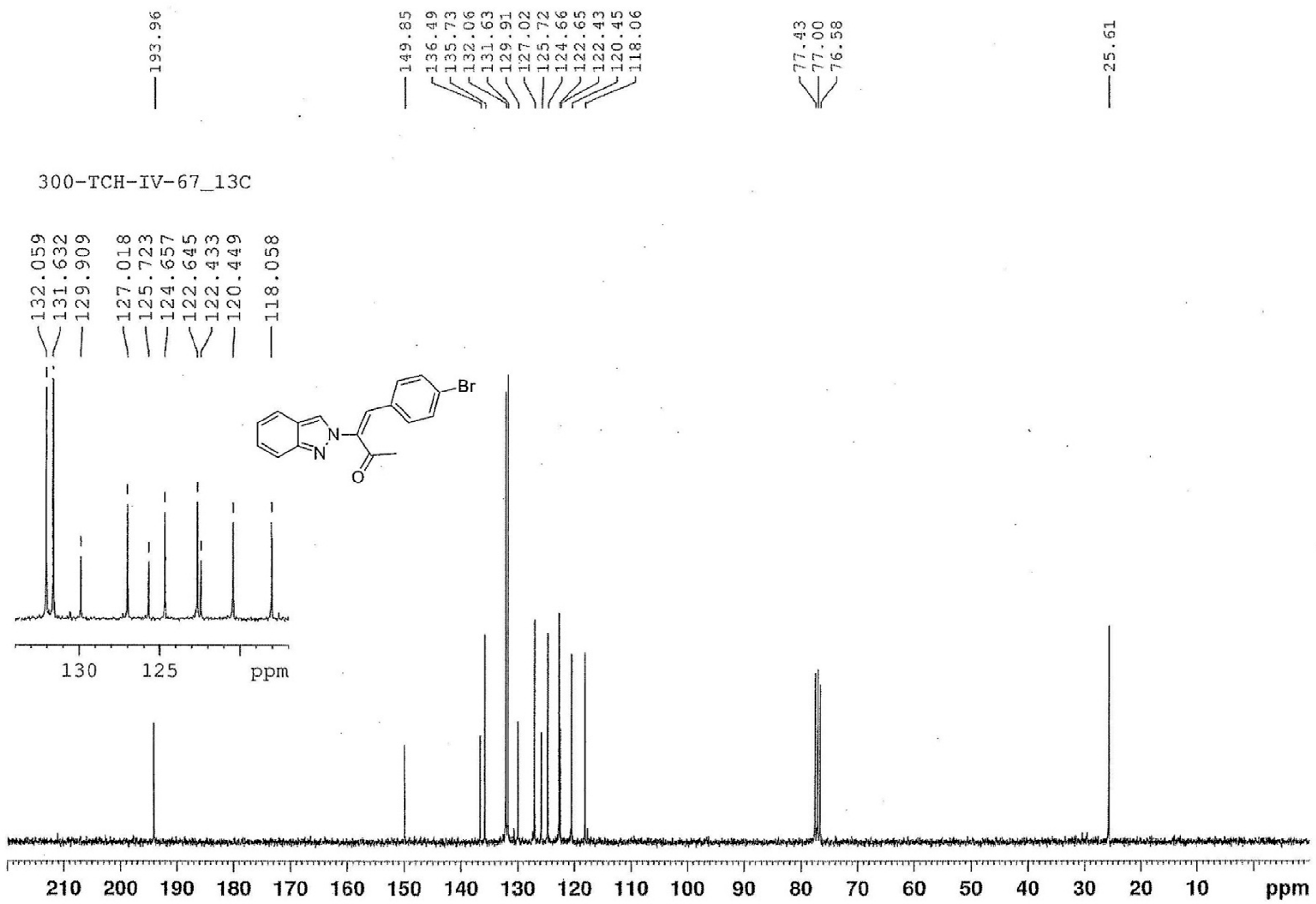

193.96
149.85
136.49
135.73
132.06
131.63
129.91
127.02
125.72
124.66
122.65
122.43
120.45
118.06
77.43
77.00
76.58
25.61
300-TCH-IV-67_13C
132.059
131.632
129.909
127.018
125.723
124.657
122.645
122.433
120.449
118.058
130
125
ppm
Br
N
N
O
210
200
190
180
170
160
150
140
130
120
110
100
90
80
70
60
50
40
30
20
10
ppm

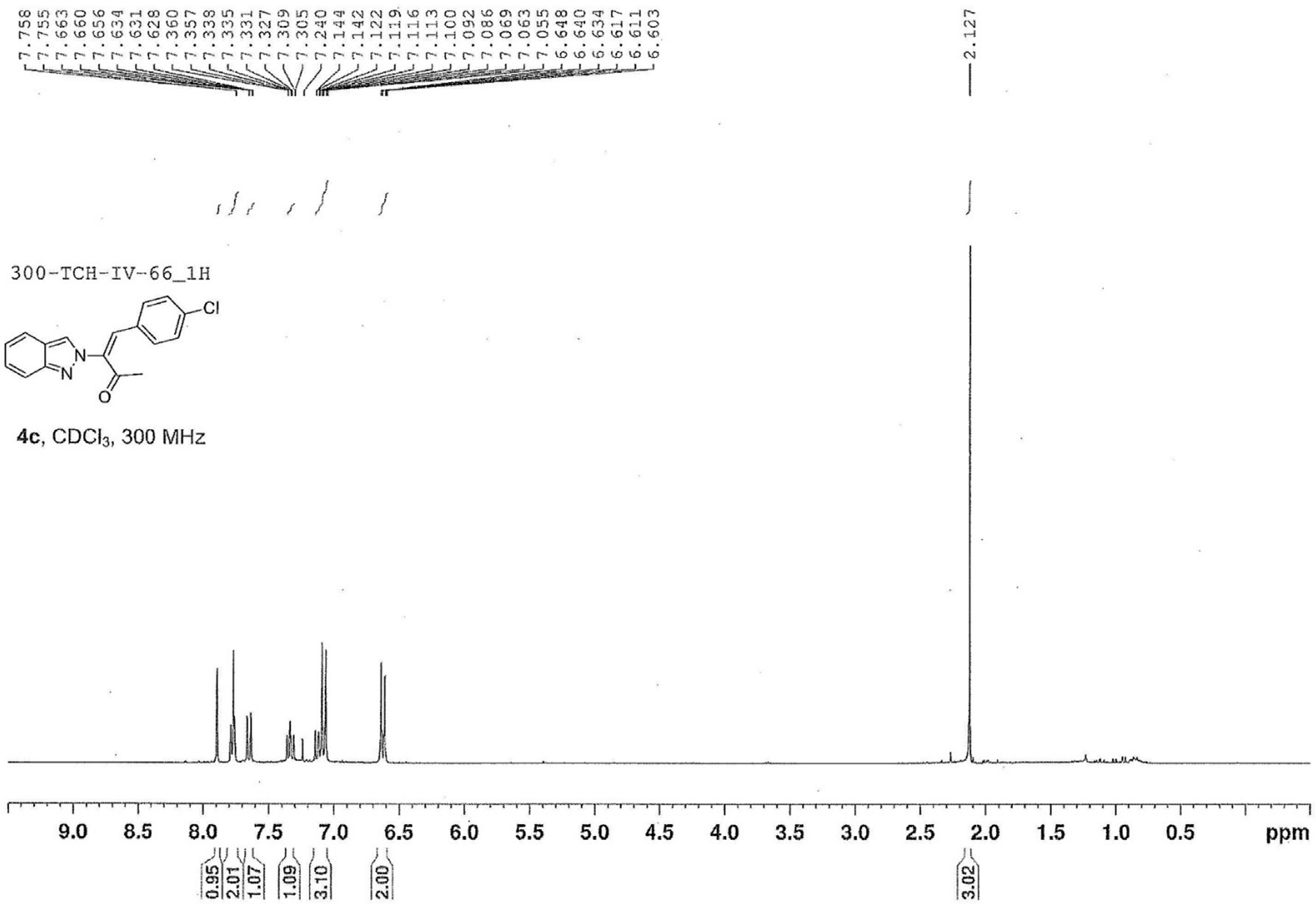
7.758
7.755
7.663
7.660
7.656
7.634
7.631
7.628
7.360
7.357
7.338
7.335
7.331
7.327
7.309
7.305
7.240
7.144
7.142
7.122
7.119
7.116
7.113
7.100
7.092
7.086
7.069
7.063
7.055
6.648
6.640
6.634
6.617
6.611
6.603
2.127
300-TCH-IV-66_1H
Cl
N
N
O
4c, CDCl3, 300 MHz
9.0 8.5 8.0 7.5 7.0 6.5 6.0 5.5 5.0 4.5 4.0 3.5 3.0 2.5 2.0 1.5 1.0 0.5 ppm
0.95
2.01
1.07
1.09
3.10
2.00
3.02

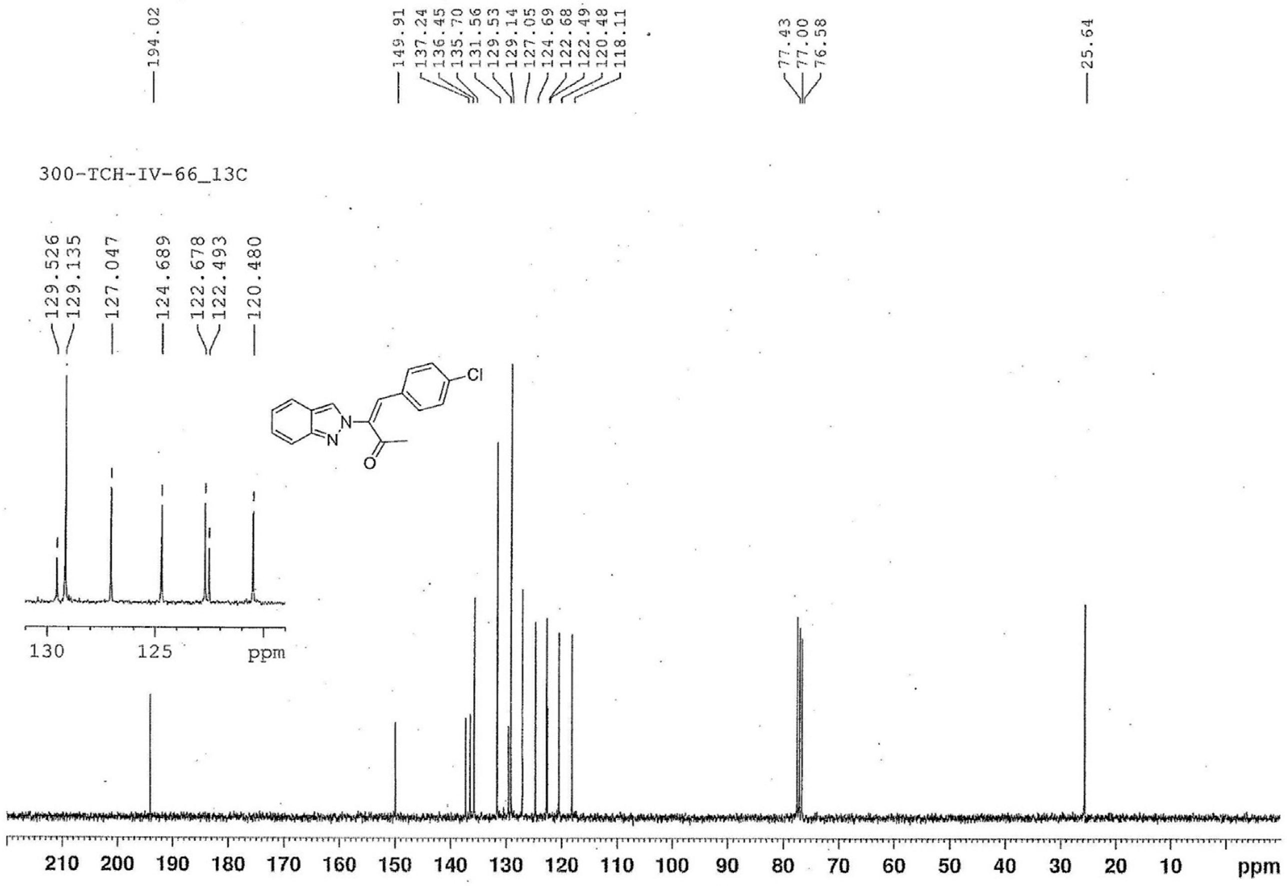
194.02
149.91
137.24
136.45
135.70
131.56
129.53
129.14
127.05
124.69
122.68
122.49
120.48
118.11
77.43
77.00
76.58
25.64
300-TCH-IV-66_13C
129.526
129.135
127.047
124.689
122.678
122.493
120.480
130
125
ppm
Cl
N
O
210 200 190 180 170 160 150 140 130 120 110 100 90 80 70 60 50 40 30 20 10 ppm

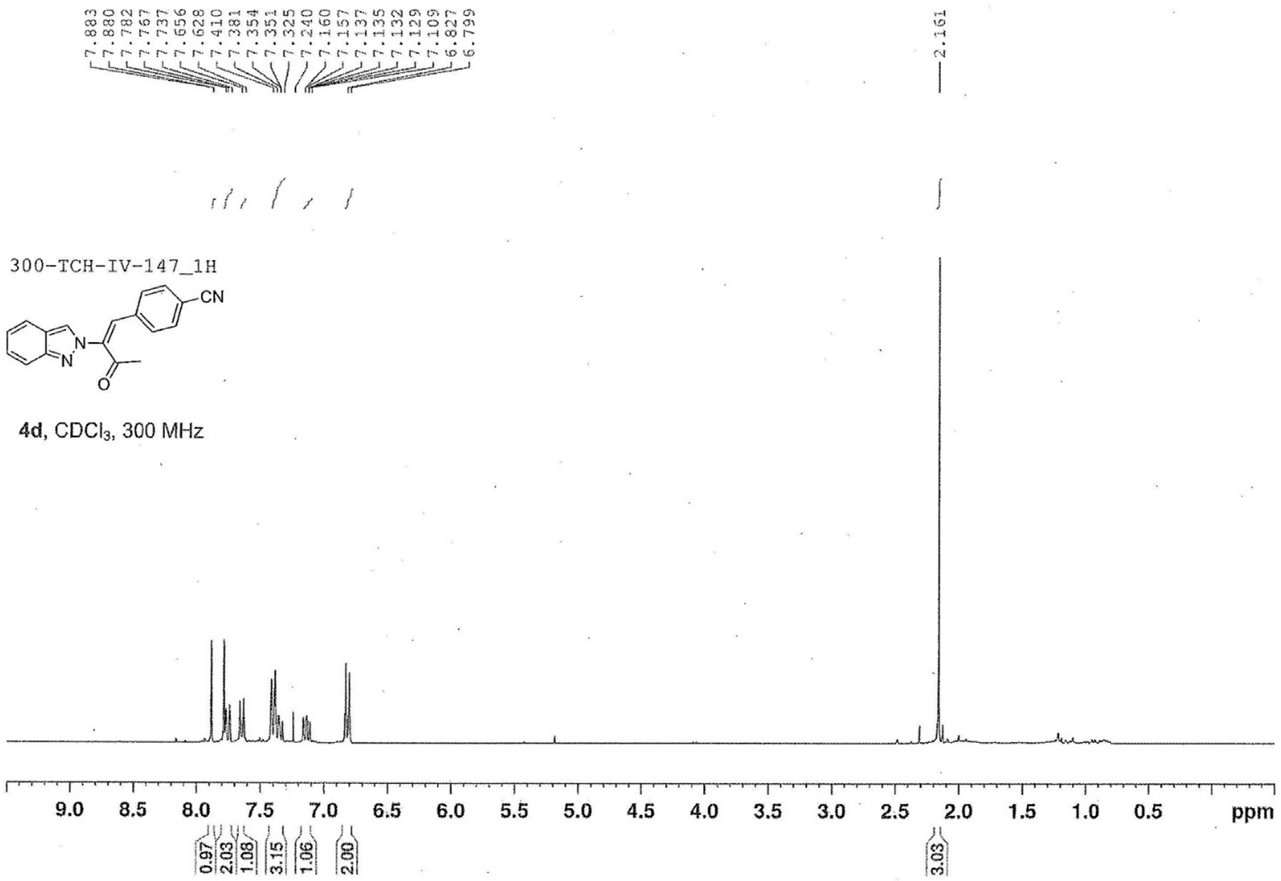
7.883
7.880
7.782
7.767
7.737
7.656
7.628
7.410
7.381
7.354
7.351
7.325
7.240
7.160
7.157
7.137
7.135
7.132
7.129
7.109
6.827
6.799
2.161
300-TCH-IV-147_1H
CN
N
N
O
4d, $CDCl_3$, 300 MHz
9.0
8.5
8.0
7.5
7.0
6.5
6.0
5.5
5.0
4.5
4.0
3.5
3.0
2.5
2.0
1.5
1.0
0.5
ppm
0.97
2.03
1.08
3.15
1.06
2.00
3.03

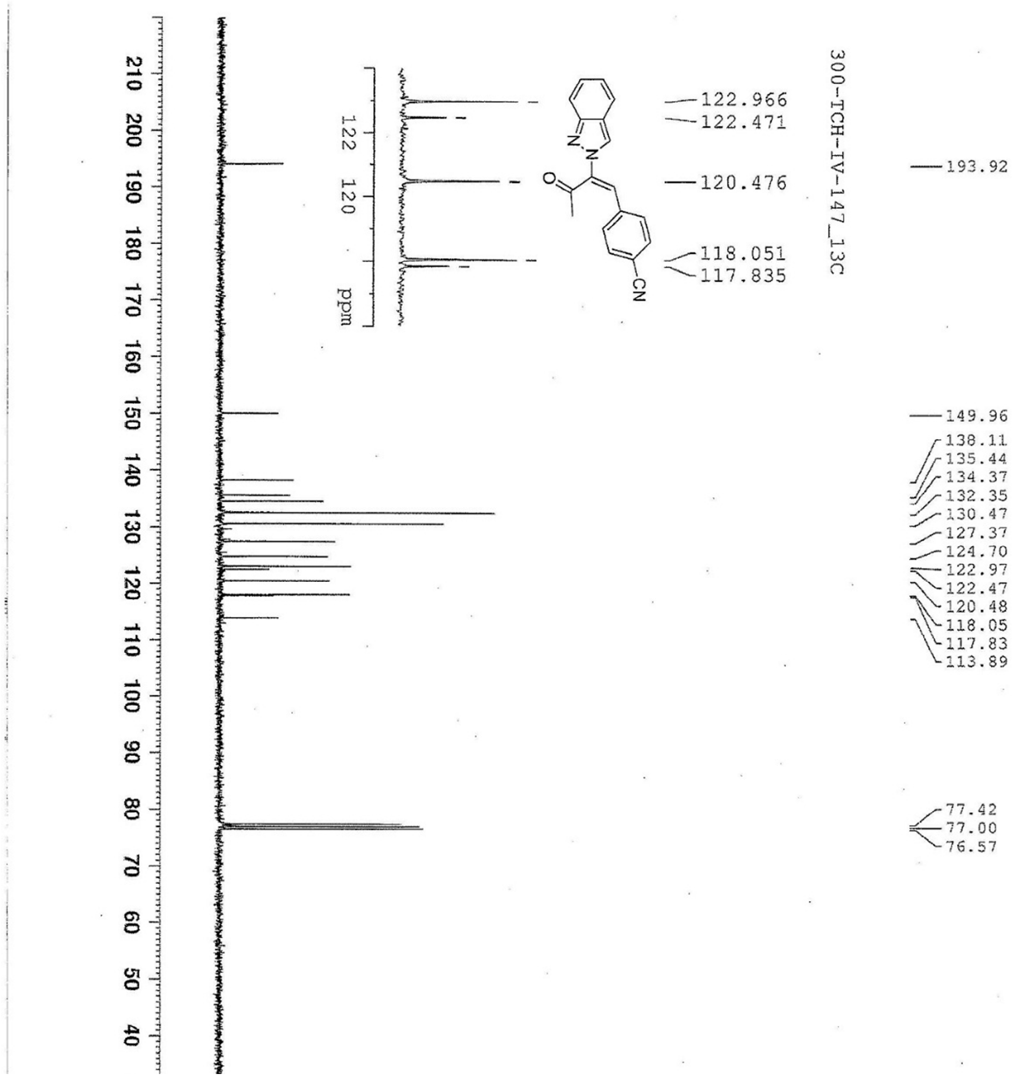
300-TCH-IV-147_13C
122.966
122.471
120.476
118.051
117.835
CN
ppm
193.92
149.96
138.11
135.44
134.37
132.35
130.47
127.37
124.70
122.97
122.47
120.48
118.05
117.83
113.89
77.42
77.00
76.57

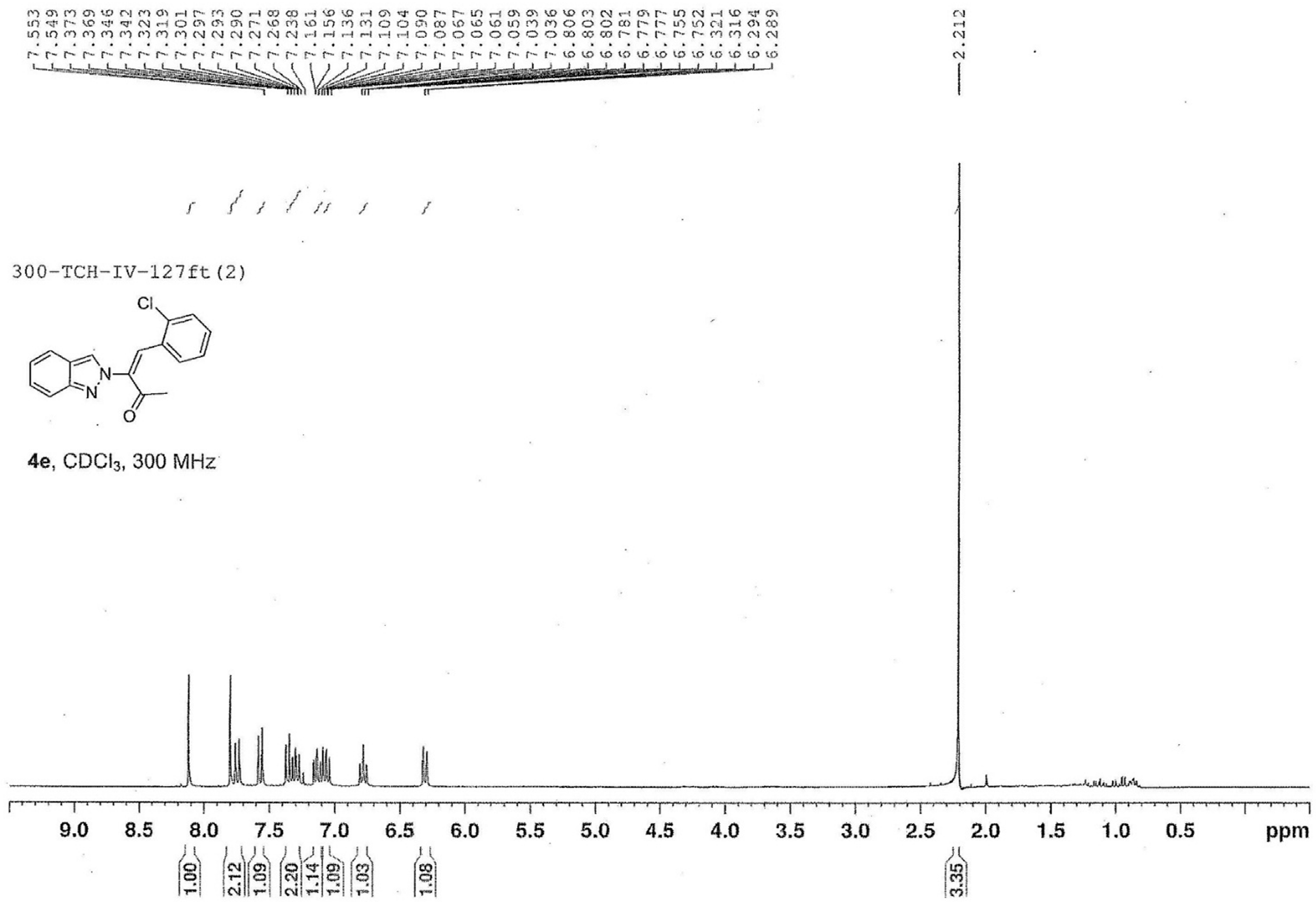
300-TCH-IV-127ft(2)
4e, $CDCl_3$, 300 MHz
ppm

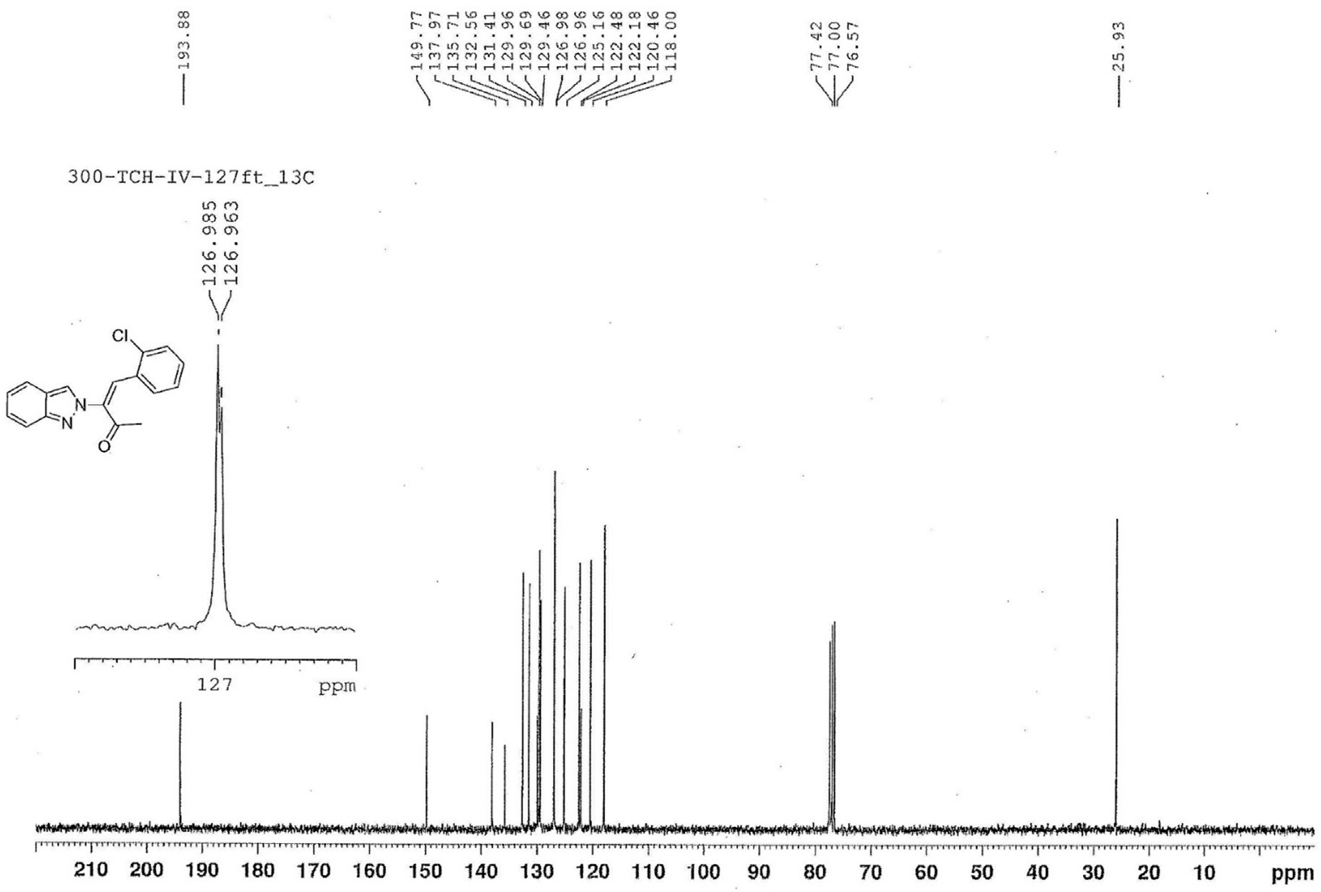
193.88
149.77
137.97
135.71
132.56
131.41
129.96
129.69
129.46
126.98
126.96
125.16
122.48
122.18
120.46
118.00
77.42
77.00
76.57
25.93
300-TCH-IV-127ft_13C
126.985
126.963
127
ppm
Cl
N
N
O
210 200 190 180 170 160 150 140 130 120 110 100 90 80 70 60 50 40 30 20 10 ppm

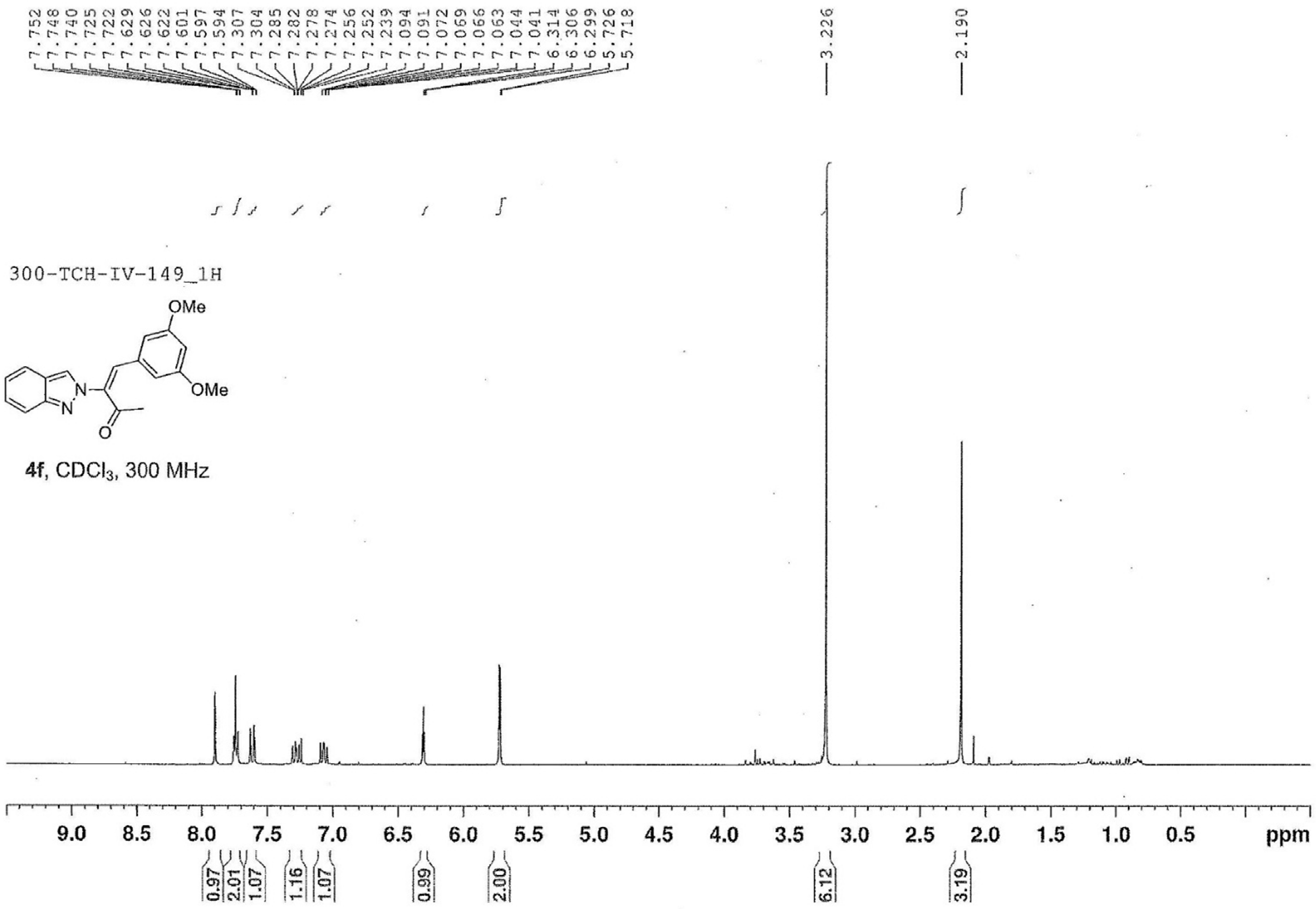

4f, $CDCl_3$, 300 MHz

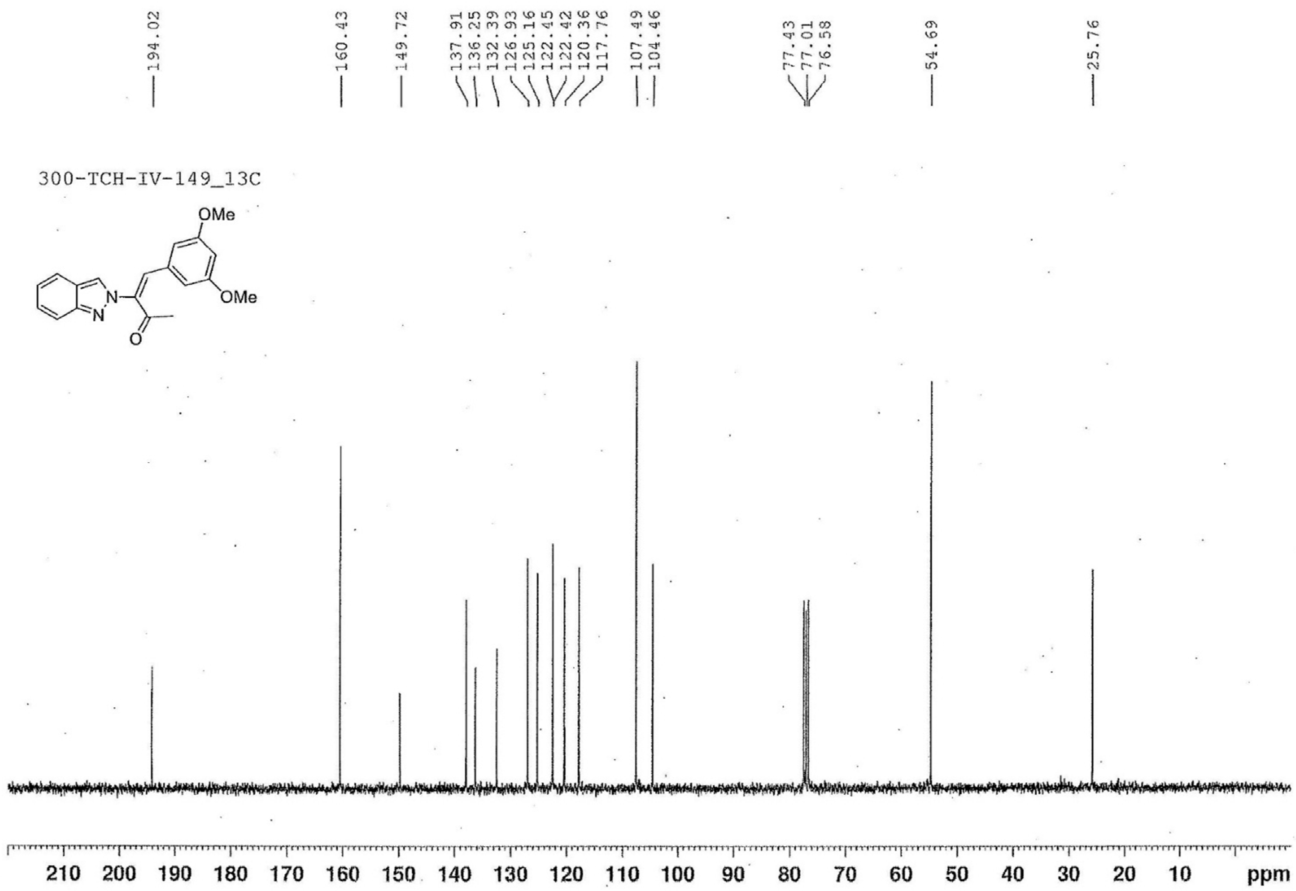
194.02
160.43
149.72
137.91
136.25
132.39
126.93
125.16
122.45
122.42
120.36
117.76
107.49
104.46
77.43
77.01
76.58
54.69
25.76
300-TCH-IV-149_13C
OMe
N
N
O
OMe
210 200 190 180 170 160 150 140 130 120 110 100 90 80 70 60 50 40 30 20 10 ppm

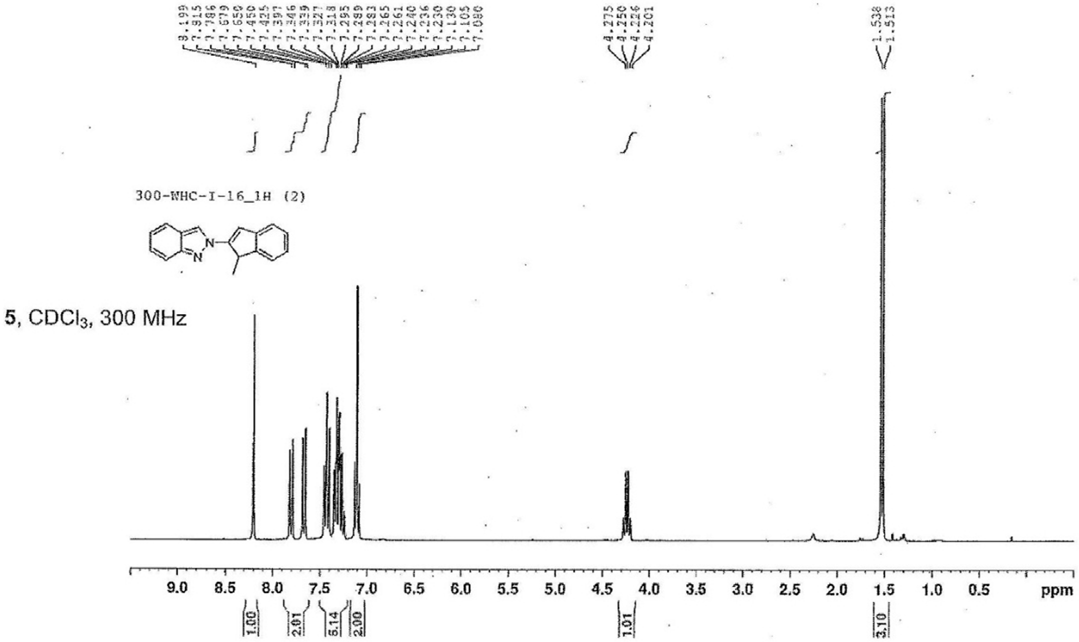
300-NHC-I-16_1H (2)
5, $CDCl_3$, 300 MHz
ppm

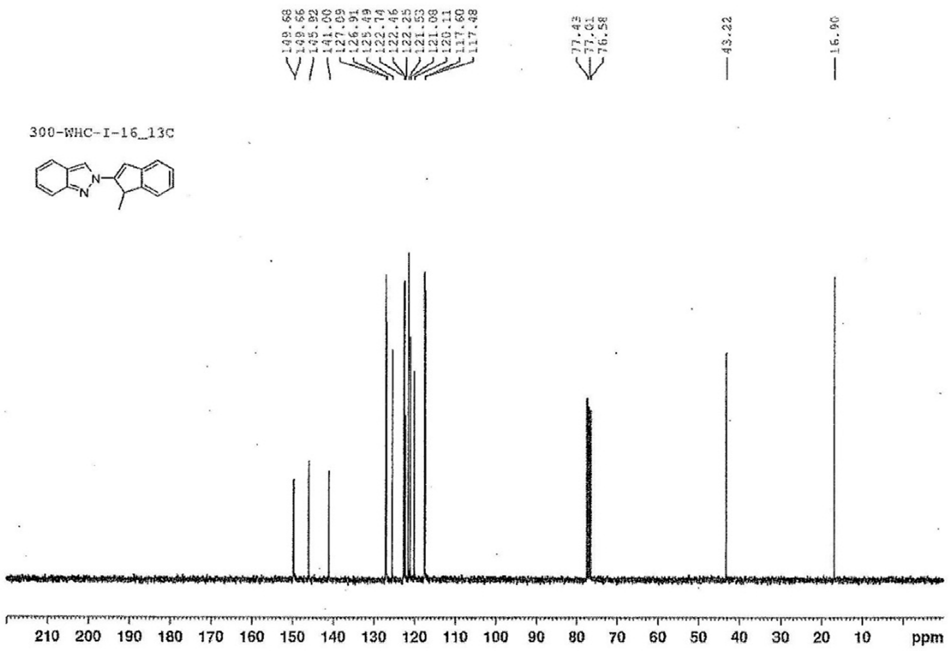

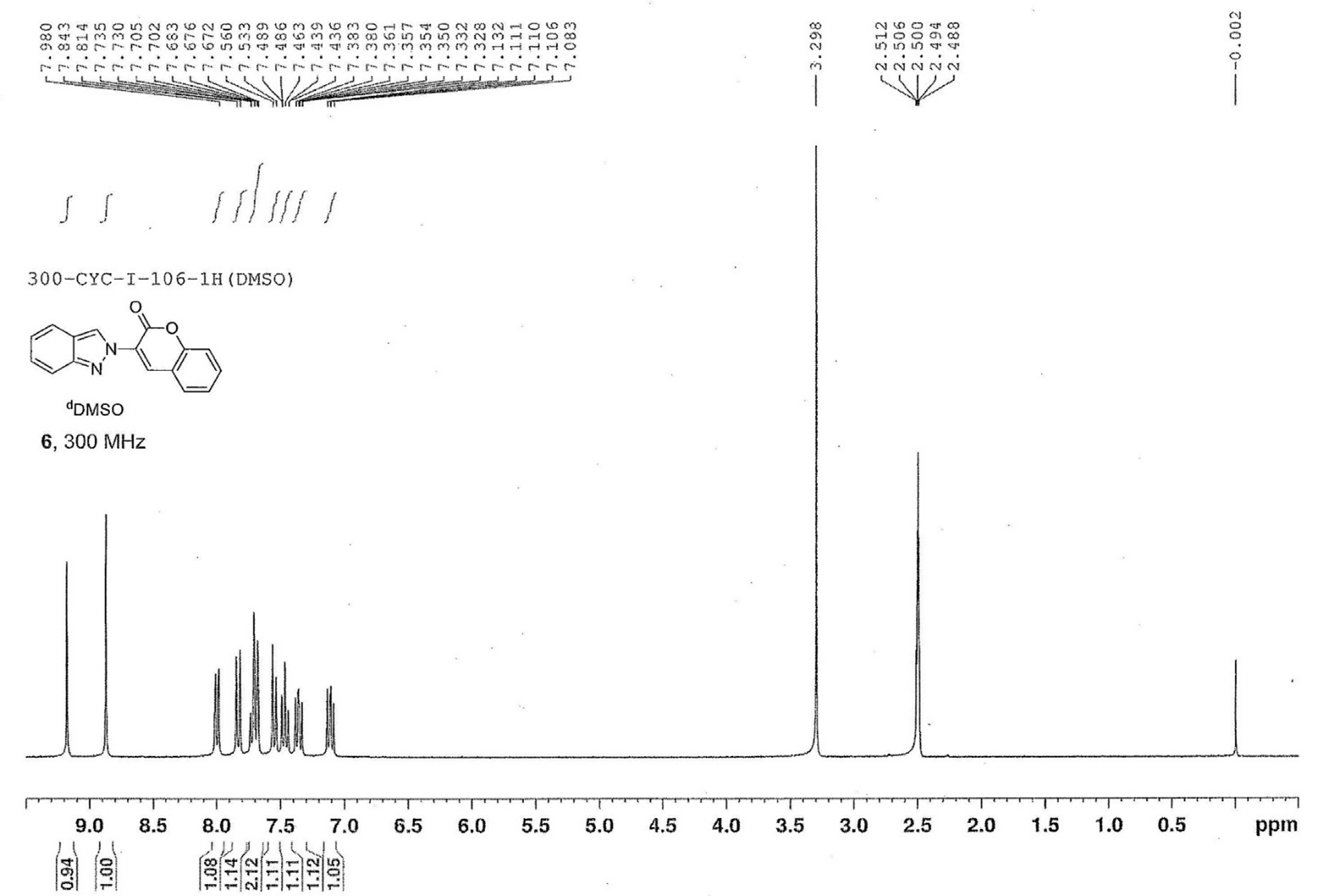
7.980
7.843
7.814
7.735
7.730
7.705
7.702
7.683
7.676
7.672
7.560
7.533
7.489
7.486
7.463
7.439
7.436
7.383
7.380
7.361
7.357
7.354
7.350
7.332
7.328
7.132
7.111
7.110
7.106
7.083
3.298
2.512
2.506
2.500
2.494
2.488
-0.002
300-CYC-I-106-1H(DMSO)
O
N
N
O
dDMSO
6, 300 MHz
9.0 8.5 8.0 7.5 7.0 6.5 6.0 5.5 5.0 4.5 4.0 3.5 3.0 2.5 2.0 1.5 1.0 0.5 ppm
0.94
1.00
1.08
1.14
2.12
1.11
1.11
1.12
1.05

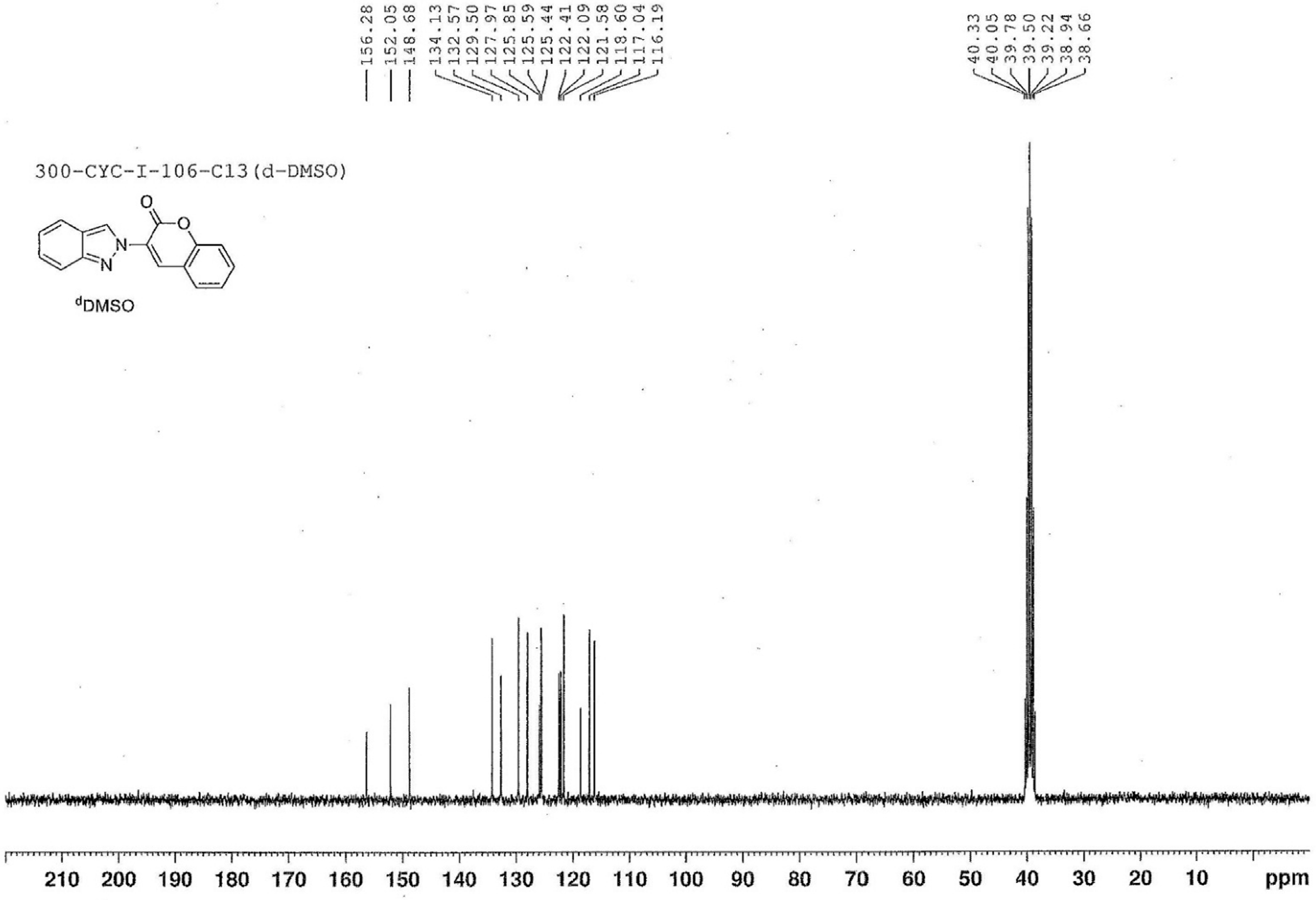
156.28
152.05
148.68
134.13
132.57
129.50
127.97
125.85
125.59
125.44
122.41
122.09
121.58
118.60
117.04
116.19
40.33
40.05
39.78
39.50
39.22
38.94
38.66
300-CYC-I-106-C13(d-DMSO)
O
N
N
O
dDMSO
210 200 190 180 170 160 150 140 130 120 110 100 90 80 70 60 50 40 30 20 10 ppm
